# Correction: Accessibility and quality of care for adults with hypertension in rural Burkina Faso: Results from a cross-sectional household survey

**DOI:** 10.1371/journal.pgph.0006093

**Published:** 2026-03-10

**Authors:** Stephanie Lacey, Maria Lisa Odland, Ali Sié, Guy Harling, Till Bärnighausen, Pascal Geldsetzer, Lisa R. Hirschhorn, Justine I. Davies

In Table 2, some headings are in the results column. Please see the correct Table 2 here.

S1 Checklist was published with comments instead of a clean version. Please view the correct S1 Checklist below.

**S1 Checklist. Inclusivity in global research.** https://doi.org/10.1371/journal.pgph.0003161.s003.

In S3 Table, here is an error in paragraph explaining results in measurement column. It should read: Overall, how well did received care meet health needs at last visit†. Please view the correct S3 Table below.

**S3 Table. Patient-centredness of care for adults with hypertension that visited a health facility in the last three months (N=250).**

In this subgroup, experiential quality was categorised as higher quality in 12.8% of participants for ease of following instructions, in 6.4% of participants for clarity of communication, in 36.4% of participants for involvement in treatment, in 16.0% of participants for trust in skills and abilities of healthcare workers, and in 49.6% of participants for opinion of medical provider knowledge and skills. Overall, 13.2% of participants had to borrow or sell something to pay for care. The mean SUDM score in this subgroup was 70.9 (11.4), range 40.0–100.0. Overall, 9.2% of participants reported higher-quality care to need being met at last visit, 39.0% of participants were very confident if they became unwell tomorrow they would receive effective treatment, and 64.3% of participants had a positive overall view of the health service.

In the S9 Table, there is an error in the description of how prevalent hypertension population defined. It should read: Model 2 Hypertension population includes those identified as hypertensive when examined by a study investigator and/or those who have previously been told they have hypertension and/or those who are currently taking treatment for hypertension, excludes participants with missing BMI data (N = 1004). Please view the correct S9 Table below.

**S9 Table. Multivariable association between sociodemographic characteristics and likelihood of progressing through the care cascade (model 2).**

In the Patient-centredness of care subsection of the Results, there is an error in the third sentence of the last paragraph. The correct sentence is: Except for the domains of clarity of communication and need met at the last visit, whereby the proportion of participants reporting higher-quality care was numerically lower in participants with a recent visit (6.4% and 9.2%, respectively), compared with participants who had a healthcare facility appointment in the more distant past; statistical testing was not done.

**Table 2:** Patient-centredness of care for adults with hypertension.

Measure	Rating	N (%)	Binary rating	N (%)
Ease of following instructions*	Very easy**	104 (11.1)	Higher quality	104 (11.1)
	Easy	692 (74.1)	Lower quality	830 (88.9)
	Fair	115 (12.3)		
	Hard	21 (2.2)		
	Very hard	2 (0.2)		
Clarity of communication*	Excellent**	57 (6.1)	Higher quality	375 (40.1)
	Very good**	318 (34.0)		
	Good	515 (55.1)	Lower quality	559 (59.9)
	Fair	41 (4.4)		
	Poor	3 (0.3)		
Involvement in treatment decisions*	Excellent**	40 (4.3)	Higher quality	254 (27.2)
	Very good**	214 (22.9)		
	Good	472 (50.5)	Lower quality	680 (72.8)
	Fair	129 (13.8)		
	Poor	79 (8.5)		
Trust in skills and abilities of healthcare worker*	Very much**	107 (11.5)	Higher quality	107 (11.5)
	Quite a bit	619 (66.3)	Lower quality	827 (88.5)
	Some	188 (20.1)		
	Very little	19 (2.0)		
	Not at all	1 (0.1)		
Opinion of medical provider knowledge and skills*	Excellent**	68 (7.3)	Higher quality	371 (39.7)
	Very good**	303 (32.4)		
	Good	514 (55.0)	Lower quality	563 (60.3)
	Fair	44 (4.7)		
	Poor	5 (0.5)		
Shared understanding and decision making* (SUDM) (Range 25.0 to 100.0)	Mean (sd)	68.5 (10.8)		
Borrowed or sold anything to pay for healthcare*	Yes	115 (12.3)		
	No	819 (87.7)		
Overall, how well did received care meet health needs at last visit^†^	Excellent**	80 (8.6)	Higher quality	415 (44.5)
	Very good**	335 (35.9)		
	Good	457 (49.0)	Lower quality	517 (55.5)
	Fair	49 (5.3)		
	Poor	11 (1.2)		
Confidence that would receive effective treatment if very sick tomorrow^†^	Very confident**	297 (31.9)	Higher quality	297 (31.9)
	Somewhat confident	567 (60.8)	Lower quality	635 (68.1)
	Not very confident	62 (6.7)		
	Not at all confident	6 (0.6)		
Overall view of national health care system^†^	Only minor changes needed to healthcare system**	576 (61.8)	Higher quality	576 (61.8)
	Major changes needed to healthcare system	324 (34.8)	Lower quality	356 (38.2)
	Need to rebuild healthcare system	32 (3.4)		

* Total population with hypertension included in the experiential quality analysis is 934 participants, this excludes those with missing visit or experiential quality data. † Total population with hypertension included in the quality care outcomes analysis is 932 participants, this excludes participants with missing visit, experiential quality, and quality outcome data. ** Indicates responses above the median value and dichotomised as ‘higher quality’ for subsequent analyses. N, number; sd, standard deviation.

**Table:** 

Measure	Rating	N (%)	Binary rating	N (%)
Ease of following instructions	Very easy*	32 (12.8)	Higher quality	32 (12.8)
	Easy	182 (72.8)	Lower quality	218 (87.2)
	Fair	29 (11.6)		
	Hard	6 (2.4)		
	Very hard	1 (0.4)		
Clarity of communication	Excellent*	16 (6.4)	Higher quality	16 (6.4)
	Very good	115 (46.0)	Lower quality	234 (93.6)
	Good	109 (43.6)		
	Fair	10 (4.0)		
	Poor	0 (0.0)		
Involvement in treatment decisions	Excellent*	16 (6.4)	Higher quality	91 (36.4)
	Very good*	75 (30.0)		
	Good	97 (38.8)	Lower quality	159 (63.6)
	Fair	41 (16.4)		
	Poor	21 (8.4)		
Trust in skills and abilities of healthcare worker	Very much*	40 (16.0)	Higher quality	40 (16.0)
	Quite a bit	163 (65.5)	Lower quality	210 (84.0)
	Some	44 (17.6)		
	Very little	2 (0.8)		
	Not at all	1 (0.4)		
Opinion of medical provider knowledge and skills	Excellent*	27 (10.8)	Higher quality	124 (49.6)
	Very good*	97 (38.8)		
	Good	119 (47.6)	Lower quality	126 (50.4)
	Fair	6 (2.4)		
	Poor	1 (0.4)		
Shared understanding and decision making (SUDM) (range 40.0 to 100.0)	Mean (sd)	70.9 (11.4)		
Borrowed or sold anything to pay for healthcare	Yes	33 (13.2)		
	No	217 (86.8)		
Overall, how well did received care meet health needs at last visit^†^	Excellent*	23 (9.2)	Higher quality	23 (9.2)
	Very good	112 (45.0)	Lower quality	226 (90.8)
	Good	96 (38.6)		
	Fair	15 (6.0)		
	Poor	3 (1.2)		
Confidence that would receive effective treatment if very sick tomorrow^†^	Very confident*	97 (39.0)	Higher quality	97 (39.0)
	Somewhat confident	138 (55.4)	Lower quality	152 (61.0)
	Not very confident	13 (5.2)		
	Not at all confident	1 (0.4)		
Overall view of national health care system^†^	Only minor changes needed to healthcare system*	160 (64.3)	Higher quality	160 (64.3)
	Major changes needed to healthcare system	81 (32.5)	Lower quality	89 (35.7)
	Need to rebuild healthcare system	8 (3.2)		

* Above median values which equate to the ‘higher quality’ binary value. ^†^ One participant excluded for missing quality outcome data. N, number; sd, standard deviation.

**Table:** 

Parameter	Group	Screened vs not		Diagnosed vs not		Treated vs not		Controlled vs not	
		POR (95% CI)	P value	POR (95% CI)	P value	POR (95% CI)	P value	POR (95% CI)	P value
Gender	Male	Referent	–	Referent	–	Referent	–	Referent	–
	Female	1.66 (1.21 to 2.28)	0.002	1.51 (1.02 to 2.25)	0.041	1.57 (0.96 to 2.56)	0.073	1.61 (0.69 to 3.78)	0.273
Age*		1.01 (1.00 to 1.03)	0.095	1.00 (0.99 to 1.02)	0.615	1.04 (1.01 to 1.06)	0.001	0.99 (0.95 to 1.02)	0.458
Education level	No formal education	Referent	–	Referent	–	Referent	–	Referent	–
	Any education	1.68 (1.08 to 2.60)	0.020	0.86 (0.54 to 1.35)	0.511	1.02 (0.58 to 1.80)	0.933	1.36 (0.53 to 3.46)	0.520
Marital status	Single/ divorced/ widowed	Referent	–	Referent	–	Referent	–	Referent	–
	Married/ cohabiting	1.62 (1.12 to 2.33)	0.010	0.99 (0.62 to 1.59)	0.967	0.87 (0.51 to 1.50)	0.622	0.77 (0.32 to 1.88)	0.569
Wealth quintile	1	Referent	–	Referent	–	Referent	–	Referent	–
	2	1.45 (0.94 to 2.24)	0.090	1.35 (0.69 to 2.61)	0.379	0.56 (0.24 to 1.31)	0.185	1.76 (0.41 to 7.59)	0.451
	3	1.76 (1.15 to 2.71)	0.010	1.12 (0.60 to 2.10)	0.718	0.45 (0.20 to 1.04)	0.061	0.48 (0.11 to 1.99)	0.311
	4	2.71 (1.78 to 4.14)	<0.001	1.28 (0.71 to 2.31)	0.412	0.80 (0.38 to 1.66)	0.550	0.37 (0.11 to 1.25)	0.110
	5	5.77 (3.57 to 9.31)	<0.001	1.41 (0.78 to 2.54)	0.252	1.06 (0.52 to 2.17)	0.878	0.96 (0.30 to 3.00)	0.940
BMI	<18.5 kg/m^2^	Referent	–	Referent	–	Referent	–	Referent	–
	18.5–24.9 kg/m^2^	1.36 (0.92 to 2.02)	0.128	0.92 (0.52 to 1.63)	0.771	1.63 (0.76 to 3.50)	0.210	2.55 (0.64 to 10.22)	0.185
	25–29.9 kg/m^2^	1.54 (0.94 to 2.52)	0.090	1.32 (0.67 to 2.62)	0.422	2.65 (1.13 to 6.22)	0.026	1.89 (0.42 to 8.49)	0.407
	≥30–kg/m^2^	2.91 (1.41 to 6.03)	0.004	1.44 (0.64 to 3.21)	0.375	2.44 (0.94 to 6.31)	0.066	1.17 (0.23 to 5.91)	0.850

Model 2 Hypertension population includes those identified as hypertensive when examined by a study investigator and/or those who have previously been told they have hypertension and/or those who are currently taking treatment for hypertension, excludes participants with missing BMI data (N = 1004). The denominator population at each step of the cascade includes the participants that achieved the previous step. For example, the denominator population for the analysis of the association between sociodemographic characteristics and diagnosed vs not, are the participants that were screened for hypertension in the previous step of the cascade. Prevalent hypertension population (N = 1002), screened (N = 623), diagnosed (N = 428), treated (N = 149), controlled disease (N = 68). * Age in years, adults aged ≥40 years. BMI, body mass index; CI, confidence interval; N, number; POR, prevalence odds ratio.
