# Supplementary figures and images for: Accessibility and quality of care for adults with hypertension in rural Burkina Faso: results from a cross-sectional household survey
Source: PLOS Glob Public Health. 2025 Apr 2;5(4):e0003161. doi: 10.1371/journal.pgph.0003161 (PMC11964235; doi:10.1371/journal.pgph.0003161)

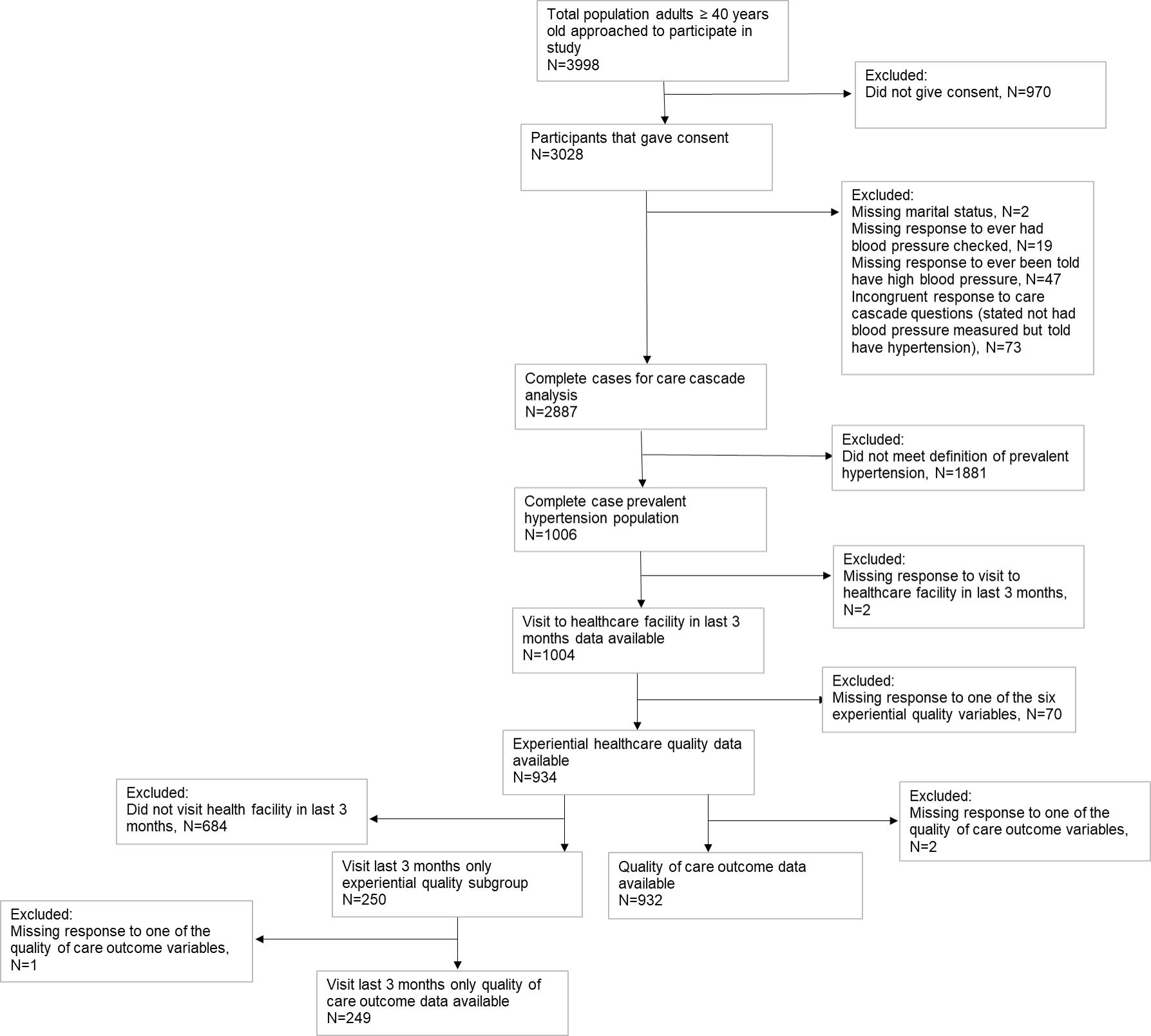

Supplement: S1 Fig — Model 1 did not include body mass index (BMI) in the multivariable analysis and thus includes participants with missing BMI data. (TIF) [file pgph.0003161.s001.tif]

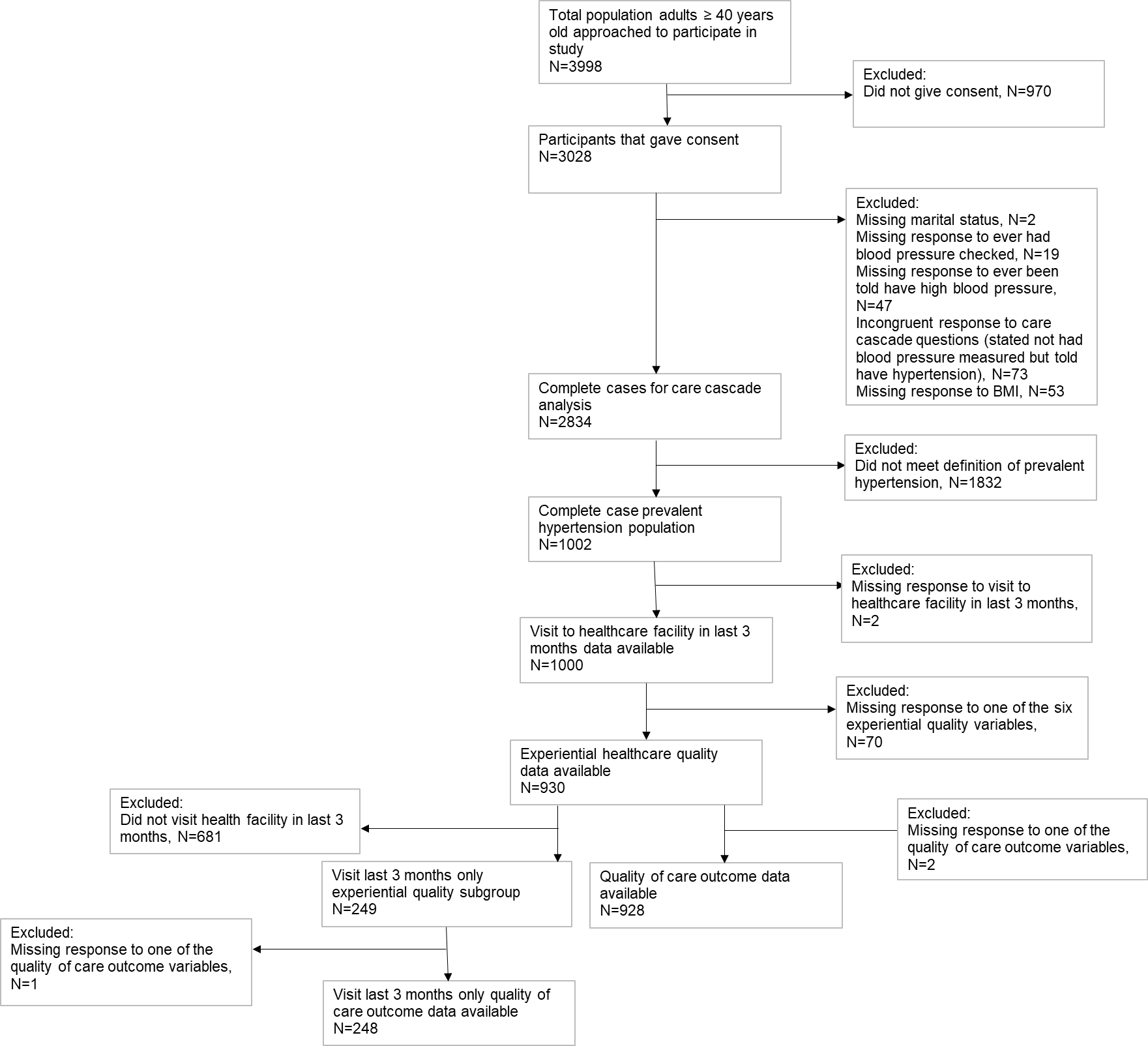

Supplement: S2 Fig — Model 2 includes body mass index (BMI) in the multivariable analysis and thus removes participants with missing BMI data. (TIF) [file pgph.0003161.s002.tif]
