# Supplementary material for: Accessibility and quality of care for adults with hypertension in rural Burkina Faso: results from a cross-sectional household survey
Source: PLOS Glob Public Health. 2025 Apr 2;5(4):e0003161. doi: 10.1371/journal.pgph.0003161 (PMC11964235; doi:10.1371/journal.pgph.0003161)
Supplement: S1 Checklist — (DOCX) [file pgph.0003161.s003.docx]

Inclusivity in global research

PLOS’ policy on inclusivity in global research aims to improve transparency in the reporting of research performed outside of researchers’ own country or community and ensures that PLOS publications reporting global research adhere to high standards for research ethics and authorship. Authors of relevant research articles may be asked to complete the questionnaire below, which outlines ethical, cultural, and scientific considerations specific to inclusivity in global research. This questionnaire may be requested when researchers have travelled to a different country to conduct research, if research uses samples collected in another country, research with Indigenous populations or their lands, or if research is on cultural artefacts. Researchers travelling to another country solely to use laboratory equipment will not normally be required to complete the questionnaire. However, the questionnaire can be requested at the journal’s discretion for any submission – if you have been requested to complete this questionnaire by the PLOS journal you submitted to, please do so.

Please complete the questionnaire below and include this as a Supporting Information file with your manuscript. Note that if your paper is accepted for publication, this checklist will be published with your article in the supporting information files. Please ensure that you reference the checklist in the main body of your manuscript. We suggest adding a subsection ‘Inclusivity in global research’ to your Methods section and adding the following sentence: “Additional information regarding the ethical, cultural, and scientific considerations specific to inclusivity in global research is included in the Supporting Information (SX Checklist)”

The questions have been designed to be applicable to a wide range of study types, and there are subsections for both human subjects research and non-human subjects research. If any of the questions are not relevant to your research please mark them as “N/A” as appropriate.

**Ethical considerations, permits and authorship**

*This section is applicable to all research types.*

Provide details as to who granted permissions and/or consent for the study to take place in the Methods section of your manuscript. This should include the names of **all** ethics boards, governmental organizations, community leaders or other bodies that provided approval for the study. If individuals provided approval refer to these people by their role or title but do not list their name(s).

Reported on page number: 9 and 10

Ethical approval for the primary analysis was given from the Ethics Commission I (medical facility Heidelberg [S-120/2018]), the Burkina Faso Comité d’Ethique pour la Recherche en Santé (2018-4-045) and the Institutional Ethics Committee of the CRSN (2018-04). Written informed consent was provided by study participants and verbal approval was granted from village elders. The detection of undiagnosed hypertension in study participants led to a referral to an appropriate health service supported by provision of funds for transportation.

If there were any deviations from the study protocol after approval was obtained please provide details of these changes in the Methods section of your manuscript.
Did this study involve local collaborators that are residents of the country where the research was conducted or members of the community studied? If you do not have any authors from said communities, please provide an explanation for this below.

Yes - The study was done in full collaboration with the NOUNA Demographic and Health Surveilance Site which is led by the Centre de Recherche en Santé de Nouna (CSRN). CSRN is a locally run organisation, fully integrated within the national Ministry of Health, which has been collecting data from the population on an annual basis for nearly 30 years. Dr Ali Sié, a CSRN staff member is an author on the manuscript.

No deviations from the protocol were noted.

Everyone listed as an author should meet PLOS’ criteria for authorship and all individuals who meet these criteria should be included in the author byline, rather than the acknowledgements. For further information please see the journal’s Authorship Policy.

We confirm that all authors meet the PLOS criteria and are included in the author byline.

**Human subjects research (e.g. health research, medical research, cross-cultural psychology)**

Did you obtain written informed consent from a representative of the local community or region before the research took place? How did you establish who speaks for the community? Details of written informed consent obtained from study participants should be reported separately in the Methods section of your manuscript.

The overarching study was designed in collaboration between the Centre de Recherche en Santé de Nouna (CRSN) and colleagues at universities in Germany, the UK and elsewhere. Prior to research conduct, consent was obtained both from the national ethics committee and the local ethics committee of the CRSN, whose membership reflects local stakeholders. Furthermore, CRSN has an ongoing relationship with village and town sector leadership structures within their health and demographic surveillance system site. Prior to starting research in any area, CRSN colleagues contacted village leaders to inform them of the study aims and gain oral agreement for the study to take place in their village. Written consent from these leaders is not a standard part of the CRSN’s study conduct and was therefore not collected for this study.

Details of the written informed consent process for participants are separately noted in the Methods.

How did members of the local community provide input on the aims of the research investigation, its methodology, and its anticipated outcome(s)?

This was a survey to determine the health conditions of the local population. There was no input from the community in the development of the survey, beyond that provided by colleagues at the CRSN, who are locally resident. However, if intervention studies are proposed as the result of our findings, we will engage community members in discussions around these.

When engaging with the local community, how did you ensure that the informed consent documents and other materials could be understood by local stakeholders?

All documents were developed in French (the national lingua franca, spoken by educated individuals) and translated into Dioula (the main local trade language) and Moore (another common local language) by data collectors fluent in the relevant languages. Participants were read the content of the information sheets in their preferred language by data collectors; study training included practice groupwork conducting this explanation and the process was reviewed after piloting in the community.

Will the findings of the research be made available in an understandable format to stakeholders in the community where the study was conducted (e.g. via a presentation, summary report, copies of publications, etc.)? Please provide details of how this will be achieved.

We will disseminate findings to local health centers and community groups as well as district, provincial and national health authorities with the goal of informing policy makers and planners to improve health of older adults.

**Non-human subjects research using specimens/ animals collected as part of the study, or those housed in archival collections. Examples include archaeology, paleontology, botany and zoology.**

Did the permission you obtained from a local authority to perform the study include an agreement on access to outputs and benefit sharing? This may include procedures to enable fair distribution of the benefits and resources arising from the research performed. Please include any details of Prior Informed Consent and Benefit Sharing Agreements obtained. These may be required by field-specific regulations, for example the Convention on Biological Diversity (CBD) and the associated Nagoya Protocol.

N/A

If the material used in your study was imported, please A) provide the year it was imported and B) indicate whether permits were obtained to import/export the materials used, C) provide details of any permits obtained. If this information is not available, please indicate this.

N/A

If you used archival specimens, please state how the material used in your study was acquired by the institute it is held in and provide details of any permits obtained for the original excavations/ sample collection. If this information is not available, please indicate this.

N/A

How was the potential cultural significance of the materials collected in your study to local communities considered in your research design? Were Indigenous peoples and/or local researchers and institutions involved with archaeological excavations / collection of specimens? If so, please provide a description of their involvement.

N/A

If your manuscript includes photographs of human remains please indicate whether authors obtained permission from descendants or affiliated cultural communities to do so.

N/A
