# Supplementary material for: Accessibility and quality of care for adults with hypertension in rural Burkina Faso: results from a cross-sectional household survey
Source: PLOS Glob Public Health. 2025 Apr 2;5(4):e0003161. doi: 10.1371/journal.pgph.0003161 (PMC11964235; doi:10.1371/journal.pgph.0003161)
Supplement: S1 Table — (DOCX) [file pgph.0003161.s004.docx]

**S1 Table. Household asset questions used to generate wealth quintiles using the Filmer and Pritchett principal components method.**

Dividing a population into wealth quintiles (with one quintile representing a fifth [20%] of the population) is one way of understanding relative poverty in a population. Wealth quintiles can also be used to understand how equitable the distribution of other indicators is in a population according to wealth e.g., timeliness access to healthcare. The Filmer and Pritchett method for calculating wealth quintiles is a commonly used approach in low- or middle-income countries where household income data may not be readily available [1,2]. This approach determines household wealth based on asset ownership and household characteristics. Participants are asked to complete a survey containing variables deemed relevant in that setting to be indicative of wealth e.g., owns a fridge or TV. Principal Component Analysis is then used to assign a factor weight to each asset or household characteristic so that a wealth index score can be calculated for each participant. The participant wealth index scores are then ranked and divided into five equal groups (quintiles), with the first quintile representing the poorest households and fifth quintile representing the wealthiest households [2,3].

| **Household assets** | | |
| --- | --- | --- |
| W1 | What is the main source of water drunk by members of your household? | - Eau du robinet dans le logement - Eau du robinet dans la cour/concession - Eau du robinet public/borne fontaine - Eau du robinet chez le voisin - Puits à pompe ou forage - Puits protégés - Puits non protégés - Source protégée - Source non protégée - Eau de pluie - Camion citerne - Charette avec petite citerne/tonneau - Eau de surface - Eau en bouteille - Autre |
| W2 | How long does it take to get the water and return home? | - Time, in minutes |
| W3 | What type of toilet do members of your household usually use? | - Chasse d'eau - à un système d'égout - Chasse d'eau - à un système d'égout - shared - Chasse d'eau - à une fosse septique - Chasse d'eau - à une fosse septique - shared - Chasse d'eau - à des latrines - Fosses/latrines - ventilées ameliorées (VIP) - Fosses/latrines - ventilées ameliorées (VIP) - shared - Fosses/latrines - avec dalles - Fosses/latrines - avec dalles - shared - Fosses/latrines - sans dalles/trou ouvert - Fosses/latrines - sans dalles/trou ouvert - shared - Toilettes à compostage - Toilettes à compostage - shared - Pas de toilette/nature - Autre - Autre - shared |
| W4 | Do you share this toilet with other households? | - Yes/No |
| W5 | What type of fule does your household usually use for cooking? | - Gaz propane liquifié (GPL) - Charbon de bois - Bois - Repas non préparé dans le mémage - Autre |
| W6 | Do you have a separate room that you use as a kitchen? | - Yes/No |
| W7 | How many bedrooms does this household have? | - Number |
| W8 | Does your household own livestock, herds, other farm animals or poultry? | - Yes/No, if N, skip next questions if No |
| W9 | Which of the following animals does your household own: | - Yes/No |
| W10 | Vaches laitières ou taureaux? | - Yes/No |
| W11 | Autre bétail? | - Yes/No |
| W12 | Chevaux, ânes ou mules? | - Yes/No |
| W13 | Chèvres? | - Yes/No |
| W14 | Moutons? | - Yes/No |
| W15 | Poulets ou autre volaille? | - Yes/No |
| W16 | Does anyone in your household own farmland? | - Yes/No, if N, skip next question if No |
| W17 | How many hectares of farmland do household members have? | - Number |
|  | In this household, do you have: |  |
| W18 | L'électricité? | - Yes/No |
| W19 | Un poste radio? | - Yes/No |
| W20 | Une télévision? | - Yes/No |
| W21 | Un téléphone fixe? | - Yes/No |
| W22 | Un ordinateur? | - Yes/No |
| W23 | Un réfrigérateur? | - Yes/No |
| W24 | Un table? | - Yes/No |
| W25 | Des chaises? | - Yes/No |
| W26 | Une Armoire/bibliothèque? | - Yes/No |
|  | Does a member of this household own: |  |
| W27 | Une montre? | - Yes/No |
| W28 | Un téléphone portable? | - Yes/No |
| W29 | If yes, how many mobile phones in your household are currently in use? | - Number |
| W30 | How many of the mobile phones currently in use in your household are “smartphones”? | - Number |
| W31 | If so, do you pay for mobile data for any of these phones? | - Yes/No |
| W32 | Une bicyclette? | - Yes/No |
| W33 | Une motocyclette ou un scooter? | - Yes/No |
| W34 | Une charrette tirée par un animal? | - Yes/No |
| W35 | Une voiture ou une camionnette? | - Yes/No |
| W37 | Does any household member have a bank account? | - Yes/No |

**References**

1. Filmer D, Pritchett LH. Estimating wealth effects without expenditure data--or tears: an application to educational enrollments in states of India. Demography. 2001;38: 115-132.
2. Fry K, Firestone R, Chakraborty NM. Measuring Equity with Nationally Representative Wealth Quintiles. PSI. 2014. [cited 2024 Mar 01]. Available from: <https://media.psi.org/wp-content/uploads/2020/02/31012157/Wealth-Quintile-Guide.pdf>
3. Rutstein SO. Steps to constructing the new DHS Wealth Index. [cited 2024 Mar 01]. Available from: <https://dhsprogram.com/programming/wealth%20index/Steps_to_constructing_the_new_DHS_Wealth_Index.pdf>
