# Supplementary material for: Accessibility and quality of care for adults with hypertension in rural Burkina Faso: results from a cross-sectional household survey
Source: PLOS Glob Public Health. 2025 Apr 2;5(4):e0003161. doi: 10.1371/journal.pgph.0003161 (PMC11964235; doi:10.1371/journal.pgph.0003161)
Supplement: S2 Table — *Overall population with hypertension excludes two participants with missing health facility visit data. †BMI excludes the four participants with missing BMI data. BMI, body mass index; IQR, interquartile range; N, number. (DOCX) [file pgph.0003161.s005.docx]

**S2 Table.** **Description of the sociodemographic characteristics of participants with hypertension by timeliness of last attendance to a health facility.**

|  | | **Overall population with hypertension (N=1004)*** | **Attended clinic in last 3 months (N=269)** | **Did not attend clinic in last 3 months (N=735)** |
| --- | --- | --- | --- | --- |
| **Parameter** | **Group** | **N (%)** | **N (%)** | **N (%)** |
| Gender | Male | 457 (45.5) | 112 (41.6) | 346 (47.1) |
|  | Female | 547 (54.5) | 157 (58.5) | 390 (53.1) |
| Age, median (IQR) | – | 55 (47–64) | 56 (47–66) | 54 (47–63) |
| Education level | No formal education | 843 (84.0) | 226 (84.2) | 617 (83.9) |
|  | Any education | 161 (16.0) | 43 (16.0) | 118 (16.1) |
| Marital status | Single/divorced/ widowed | 291 (29.0) | 86 (32.0) | 205 (27.9) |
|  | Married/cohabiting | 713 (71.0) | 183 (68.0) | 530 (72.1) |
| Wealth quintile | 1 | 199 (19.8) | 43 (16.0) | 156 (21.1) |
|  | 2 | 164 (16.3) | 36 (13.4) | 128 (17.4) |
|  | 3 | 181 (18.0) | 54 (20.1) | 127 (17.3) |
|  | 4 | 212 (21.1) | 63 (23.4) | 149 (20.3) |
|  | 5 | 248 (24.7) | 73 (27.1) | 175 (23.8) |
| BMI^†^ | Underweight (<18.5 kg/m^2^) | 141 (14.1) | 37 (13.8) | 104 (14.2) |
|  | Normal range (18.5-24.9 kg/m^2^) | 594 (59.4) | 146 (54.5) | 448 (61.2) |
|  | Overweight (25-29.9 kg/m^2^) | 177 (17.7) | 56 (20.9) | 121 (16.5) |
|  | Obese (≥30-kg/m^2^) | 88 (8.8) | 29 (10.8) | 59 (8.1) |

*Overall population with hypertension excludes two participants with missing health facility visit data. ^†^BMI excludes the four participants with missing BMI data.

BMI, body mass index; IQR, interquartile range; N, number.
