# Supplementary material for: Accessibility and quality of care for adults with hypertension in rural Burkina Faso: results from a cross-sectional household survey
Source: PLOS Glob Public Health. 2025 Apr 2;5(4):e0003161. doi: 10.1371/journal.pgph.0003161 (PMC11964235; doi:10.1371/journal.pgph.0003161)
Supplement: S3 Table — *Above median values which equate to the ‘higher quality’ binary value. †One participant excluded for missing quality outcome data. N, number; sd, standard deviation. (DOCX) [file pgph.0003161.s006.docx]

**S3 Table. Patient-centredness of care for adults with hypertension that visited a health facility in the last three months (N=250).**

In this subgroup, experiential quality was categorised as higher quality in 12.8% of participants for ease of following instructions, in 6.4% of participants for clarity of communication, in 36.4% of participants for involvement in treatment, in 16.0% of participants for trust in skills and abilities of healthcare workers, and in 49.6% of participants for opinion of medical provider knowledge and skills. Overall, 13.2% of participants had to borrow or sell something to pay for care. The mean SUDM score in this subgroup was 70.9 (11.4), range 40.0–100.0. Overall, 9.2% of participants reported higher-quality care (excellent or very good) to need being met at last visit, 39.0% of participants were very confident if they become unwell tomorrow they would receive effective treatment, and 64.3% of participants had a positive overall view of the health service.

| **Measure** | **Rating** | **N (%)** | **Binary rating** | **N (%)** |
| --- | --- | --- | --- | --- |
| Ease of following instructions | Very easy* | 32 (12.8) | Higher quality | 32 (12.8) |
|  | Easy | 182 (72.8) | Lower quality | 218 (87.2) |
|  | Fair | 29 (11.6) |  |  |
|  | Hard | 6 (2.4) |  |  |
|  | Very hard | 1 (0.4) |  |  |
| Clarity of communication | Excellent* | 16 (6.4) | Higher quality | 16 (6.4) |
|  | Very good | 115 (46.0) | Lower quality | 234 (93.6) |
|  | Good | 109 (43.6) |  |  |
|  | Fair | 10 (4.0) |  |  |
|  | Poor | 0 (0.0) |  |  |
| Involvement in treatment decisions | Excellent* | 16 (6.4) | Higher quality | 91 (36.4) |
|  | Very good* | 75 (30.0) |  |  |
|  | Good | 97 (38.8) | Lower quality | 159 (63.6) |
|  | Fair | 41 (16.4) |  |  |
|  | Poor | 21 (8.4) |  |  |
| Trust in skills and abilities of healthcare worker | Very much* | 40 (16.0) | Higher quality | 40 (16.0) |
|  | Quite a bit | 163 (65.5) | Lower quality | 210 (84.0) |
|  | Some | 44 (17.6) |  |  |
|  | Very little | 2 (0.8) |  |  |
|  | Not at all | 1 (0.4) |  |  |
| Opinion of medical provider knowledge and skills | Excellent* | 27 (10.8) | Higher quality | 124 (49.6) |
|  | Very good* | 97 (38.8) |  |  |
|  | Good | 119 (47.6) | Lower quality | 126 (50.4) |
|  | Fair | 6 (2.4) |  |  |
|  | Poor | 1 (0.4) |  |  |
| Shared understanding and decision making (SUDM)  (range 40.0 to 100.0) | Mean (sd) | 70.9 (11.4) |  |  |
| Borrowed or sold anything to pay for healthcare | Yes | 33 (13.2) |  |  |
|  | No | 217 (86.8) |  |  |
| Overall, how will did received care meet health needs at last visit^†^ | Excellent* | 23 (9.2) | Higher quality | 23 (9.2) |
|  | Very good | 112 (45.0) | Lower quality | 226 (90.8) |
|  | Good | 96 (38.6) |  |  |
|  | Fair | 15 (6.0) |  |  |
|  | Poor | 3 (1.2) |  |  |
| Confidence that would receive effective treatment if very sick tomorrow^†^ | Very confident* | 97 (39.0) | Higher quality | 97 (39.0) |
|  | Somewhat confident | 138 (55.4) | Lower quality | 152 (61.0) |
|  | Not very confident | 13 (5.2) |  |  |
|  | Not at all confident | 1 (0.4) |  |  |
| Overall view of national health care system^†^ | Only minor changes needed to healthcare system* | 160 (64.3) | Higher quality | 160 (64.3) |
|  | Major changes needed to healthcare system | 81 (32.5) | Lower quality | 89 (35.7) |
|  | Need to rebuild healthcare system | 8 (3.2) |  |  |

*Above median values which equate to the ‘higher quality’ binary value. ^†^One participant excluded for missing quality outcome data. N, number; sd, standard deviation.
