# Supplementary material for: Accessibility and quality of care for adults with hypertension in rural Burkina Faso: results from a cross-sectional household survey
Source: PLOS Glob Public Health. 2025 Apr 2;5(4):e0003161. doi: 10.1371/journal.pgph.0003161 (PMC11964235; doi:10.1371/journal.pgph.0003161)
Supplement: S4 Table — Model 1, prevalent hypertension population (N=934) excludes participants with missing visit or experiential quality data. Model 2 consists of the same population as model 1 except the four participants with missing body mass index data were removed. *Age in years, adults aged ≥40 years. BMI, body mass index; CI, confidence interval; N, number; POR, prevalence odds ratio. (DOCX) [file pgph.0003161.s007.docx]

**S4 Table.** **Multivariable regression to determine the association between participant characteristics and experience of care in the prevalent hypertension population.**

| **Parameter** | **Group** | **Model 1 (N=934)** | | | | | |
| --- | --- | --- | --- | --- | --- | --- | --- |
|  |  | **Ease of following instructions** | | **Clarity of communication** | | **Involvement in treatment decisions** | |
|  |  | **POR (95% CI)** | **P value** | **POR (95% CI)** | **P value** | **POR (95% CI)** | **P value** |
| Gender | Male | Referent | – | Referent | – | Referent | – |
|  | Female | 0.91 (0.58 to 1.45) | 0.704 | **0.69 (0.51 to 0.93)** | **0.014** | 0.89 (0.64 to 1.24) | 0.501 |
| Age* | – | 1.00 (0.97 to 1.02) | 0.661 | 1.00 (0.99 to 1.01) | 0.986 | **1.02 (1.00 to 1.03)** | **0.021** |
| Education level | No formal education | Referent | – | Referent | – | Referent | – |
|  | Any education | **1.79 (1.07 to 2.99)** | **0.026** | 0.78 (0.54 to 1.14) | 0.205 | 0.69 (0.45 to 1.06) | 0.088 |
| Marital status | Single/divorced/ widowed | Referent | – | Referent | – | Referent | – |
|  | Married/cohabiting | 1.19 (0.66 to 2.13) | 0.562 | 1.02 (0.71 to 1.47) | 0.902 | 1.18 (0.79 to 1.77) | 0.410 |
| Wealth quintile | 1 | Referent | – | Referent | – | Referent | – |
|  | 2 | 1.27 (0.60 to 2.67) | 0.536 | 0.92 (0.58 to 1.46) | 0.730 | 0.97 (0.57 to 1.67) | 0.926 |
|  | 3 | 1.14 (0.54 to 2.38) | 0.733 | 1.28 (0.82 to 1.99) | 0.277 | 1.25 (0.75 to 2.07) | 0.387 |
|  | 4 | 1.14 (0.56 to 2.32) | 0.709 | 1.01 (0.66 to 1.55) | 0.972 | 1.60 (1.00 to 2.58) | 0.052 |
|  | 5 | 1.26 (0.63 to 2.51) | 0.512 | 1.33 (0.87 to 2.03) | 0.181 | **1.88 (1.18 to 3.01)** | **0.008** |
| **Parameter** | **Group** | **Trust in skills and abilities of healthcare worker** | | **Opinion of medical providers knowledge and skills** | | **Borrowed or sold anything to pay for healthcare** | |
|  |  | **POR (95% CI)** | **P value** | **POR (95% CI)** | **P value** | **POR (95% CI)** | **P value** |
| Gender | Male | Referent | – | Referent | – | Referent | – |
|  | Female | 0.87 (0.56 to 1.37) | 0.558 | 0.83 (0.62 to 1.11) | 0.215 | 0.74 (0.47 to 1.15) | 0.183 |
| Age* | – | 0.99 (0.97 to 1.01) | 0.275 | 1.00 (0.99 to 1.01) | 0.827 | 0.99 (0.98 to 1.01) | 0.596 |
| Education level | No formal education | Referent | – | Referent | – | Referent | – |
|  | Any education | 0.99 (0.57 to 1.75) | 0.985 | 1.03 (0.71 to 1.49) | 0.894 | 0.55 (0.28 to 1.08) | 0.084 |
| Marital status | Single/divorced/ widowed | Referent | – | Referent | – | Referent | – |
|  | Married/cohabiting | 1.09 (0.62 to 1.92) | 0.759 | 1.00 (0.70 to 1.43) | 0.997 | 0.99 (0.58 to 1.69) | 0.971 |
| Wealth quintile | 1 | Referent | – | Referent | – | Referent | – |
|  | 2 | 0.66 (0.32 to 1.36) | 0.263 | 1.21 (0.77 to 1.92) | 0.407 | 0.72 (0.40 to 1.28) | 0.258 |
|  | 3 | 0.84 (0.43 to 1.63) | 0.602 | 1.30 (0.84 to 2.03) | 0.240 | **0.39 (0.21 to 0.74)** | **0.004** |
|  | 4 | 0.86 (0.46 to 1.63) | 0.650 | 1.02 (0.66 to 1.57) | 0.937 | **0.32 (0.17 to 0.61)** | **<0.001** |
|  | 5 | 0.79 (0.42 to 1.49) | 0.465 | 1.23 (0.81 to 1.88) | 0.332 | **0.36 (0.20 to 0.67)** | **0.001** |
| **Parameter** | **Group** | **Model 2 (N=930)** | | | | | |
|  |  | **Ease of following instructions** | | **Clarity of communication** | | **Involvement in treatment decisions** | |
|  |  | **POR (95% CI)** | **P value** | **POR (95% CI)** | **P value** | **POR (95% CI)** | **P value** |
| Gender | Male | Referent | – | Referent | – | Referent | – |
|  | Female | 0.99 (0.62 to 1.59) | 0.980 | **0.67 (0.49 to 0.90)** | **0.009** | 0.88 (0.63 to 1.23) | 0.452 |
| Age* | – | 0.99 (0.97 to 1.01) | 0.447 | 1.00 (0.99 to 1.01) | 0.991 | **1.02 (1.00 to 1.03)** | **0.022** |
| Education level | No formal education | Referent | – | Referent | – | Referent | – |
|  | Any education | **1.73 (1.02 to 2.92)** | **0.042** | 0.76 (0.52 to 1.12) | 0.163 | 0.66 (0.43 to 1.02) | 0.064 |
| Marital status | Single/divorced/ widowed | Referent | – | Referent | – | Referent | – |
|  | Married/cohabiting | 1.18 (0.65 to 2.13) | 0.583 | 1.01 (0.71 to 1.46) | 0.943 | 1.17 (0.79 to 1.76) | 0.433 |
| Wealth quintile | 1 | Referent | – | Referent | – | Referent | – |
|  | 2 | 1.34 (0.63 to 2.83) | 0.448 | 0.89 (0.56 to 1.42) | 0.635 | 0.95 (0.56 to 1.63) | 0.858 |
|  | 3 | 1.17 (0.55 to 2.49) | 0.679 | 1.25 (0.80 to 1.95) | 0.331 | 1.22 (0.74 to 2.04) | 0.436 |
|  | 4 | 1.26 (0.62 to 2.58) | 0.525 | 0.97 (0.62 to 1.49) | 0.877 | 1.56 (0.96 to 2.52) | 0.073 |
|  | 5 | 1.50 (0.73 to 3.07) | 0.266 | 1.22 (0.78 to 1.90) | 0.378 | **1.77 (1.08 to 2.88)** | **0.023** |
| BMI | Underweight (<18.5 kg/m^2^) | Referent | – | Referent | – | Referent | – |
|  | Normal range (18.5-25 kg/m^2^) | 0.70 (0.38 to 1.32) | 0.274 | 0.89 (0.58 to 1.36) | 0.590 | 0.93 (0.58 to 1.49) | 0.766 |
|  | Overweight (25-29.9 kg/m^2^) | 0.62 (0.29 to 1.33) | 0.219 | 1.25 (0.76 to 2.06) | 0.383 | 1.24 (0.72 to 2.14) | 0.445 |
|  | Obese (≥30-kg/m^2^) | 0.41 (0.15 to 1.13) | 0.085 | 1.19 (0.64 to 2.19) | 0.585 | 1.10 (0.56 to 2.15) | 0.790 |
| **Parameter** | **Group** | **Trust in skills and abilities of healthcare worker** | | **Opinion of medical providers knowledge and skills** | | **Borrowed or sold anything to pay for healthcare** | |
|  |  | **POR (95% CI)** | **P value** | **POR (95% CI)** | **P value** | **POR (95% CI)** | **P value** |
| Gender | Male | Referent | – | Referent | – | Referent | – |
|  | Female | 0.84 (0.53 to 1.34) | 0.472 | 0.81 (0.60 to 1.09) | 0.161 | 0.71 (0.45 to 1.11) | 0.136 |
| Age* | – | 0.99 (0.97 to 1.01) | 0.241 | 1.00 (0.99 to 1.01) | 0.989 | 0.99 (0.98 to 1.02) | 0.761 |
| Education level | No formal education | Referent | – | Referent | – | Referent | – |
|  | Any education | 0.93 (0.52 to 1.66) | 0.797 | 0.97 (0.66 to 1.41) | 0.858 | 0.54 (0.27 to 1.06) | 0.075 |
| Marital status | Single/divorced/ widowed | Referent | – | Referent | – | Referent | – |
|  | Married/cohabiting | 1.06 (0.60 to 1.87) | 0.850 | 0.99 (0.69 to 1.42) | 0.971 | 0.99 (0.58 to 1.69) | 0.976 |
| Wealth quintile | 1 | Referent | – | Referent | – | Referent | – |
|  | 2 | 0.63 (0.31 to 1.32) | 0.222 | 1.16 (0.72 to 1.84) | 0.522 | 0.68 (0.38 to 1.22) | 0.192 |
|  | 3 | 0.82 (0.42 to 1.60) | 0.552 | 1.22 (0.78 to 1.91) | 0.391 | **0.36 (0.19 to 0.70)** | **0.002** |
|  | 4 | 0.82 (0.43 to 1.57) | 0.556 | 0.96 (0.62 to 1.48) | 0.842 | **0.29 (0.15 to 0.56)** | **<0.001** |
|  | 5 | 0.70 (0.35 to 1.38) | 0.299 | 1.12 (0.71 to 1.72) | 0.659 | **0.31 (0.16 to 0.60)** | **0.001** |
| BMI | Underweight (<18.5 kg/m^2^) | Referent | – | Referent | – | Referent | – |
|  | Normal range (18.5-25 kg/m^2^) | 0.76 (0.40 to 1.45) | 0.401 | 1.14 (0.74 to 1.75) | 0.541 | 1.40 (0.74 to 2.65) | 0.295 |
|  | Overweight (25-29.9 kg/m^2^) | 1.52 (0.74 to 3.13) | 0.254 | 1.52 (0.92 to 2.52) | 0.101 | 1.49 (0.69 to 3.22) | 0.309 |
|  | Obese (≥30-kg/m^2^) | 0.92 (0.35 to 2.40) | 0.864 | 1.47 (0.79 to 2.73) | 0.219 | 2.06 (0.80 to 5.34) | 0.135 |

Model 1, prevalent hypertension population (N=934) excludes participants with missing visit or experiential quality data. Model 2 consists of the same population as model 1 except the four participants with missing body mass index data were removed. *Age in years, adults aged ≥40 years. BMI, body mass index; CI, confidence interval; N, number; POR, prevalence odds ratio.
