# Supplementary material for: Accessibility and quality of care for adults with hypertension in rural Burkina Faso: results from a cross-sectional household survey
Source: PLOS Glob Public Health. 2025 Apr 2;5(4):e0003161. doi: 10.1371/journal.pgph.0003161 (PMC11964235; doi:10.1371/journal.pgph.0003161)
Supplement: S5 Table — Model 1, prevalent hypertension population (N=934) excludes participants with missing visit or experiential quality data. Model 2 consists of the same population as model 1 except the four participants with missing body mass index data were removed. *Age in years, adults aged ≥40 years. BMI, body mass index; CI, confidence interval; N, number. (DOCX) [file pgph.0003161.s008.docx]

**S5 Table. Multivariable regression to determine the association between shared understanding and decision making (SUDM) and participant characteristics.**

|  | | **Model 1 (N=934)** | | **Model 2 (N=930)** | |
| --- | --- | --- | --- | --- | --- |
| **Parameter** | **Group** | **Multivariable analysis coefficient (95% CI)** | **P value** | **Multivariable analysis coefficient (95% CI)** | **P value** |
| Gender | Male | Referent | – | Referent | – |
|  | Female | -0.01 (-0.03 to 0.00) | 0.107 | -0.02 (-0.03 to 0.00) | 0.061 |
| Age* | – | 0.00 (-0.00 to 0.00) | 0.762 | 0.00 (-0.00 to 0.00) | 0.726 |
| Education level | No formal education | Referent | – | Referent | – |
|  | Any education | -0.00 (-0.02 to 0.01) | 0.632 | -0.01 (-0.03 to 0.01) | 0.415 |
| Marital status | Single/ | Referent | – | Referent | – |
|  | divorced/ widowed |  |  |  |  |
|  | Married/ | 0.00 (-0.02 to 0.02) | 0.724 | 0.00 (-0.02 to 0.02) | 0.758 |
|  | cohabiting |  |  |  |  |
| Wealth quintile | 1 | Referent | – | Referent | – |
|  | 2 | -0.01 (-0.03 to 0.01) | 0.433 | -0.01 (-0.04 to 0.01) | 0.327 |
|  | 3 | 0.01 (-0.02 to 0.03) | 0.593 | 0.00 (-0.02 to 0.03) | 0.761 |
|  | 4 | 0.00 (-0.02 to 0.02) | 0.864 | -0.00 (-0.02 to 0.02) | 0.905 |
|  | 5 | 0.01 (-0.01 to 0.04) | 0.249 | 0.01 (-0.02 to 0.03) | 0.628 |
| BMI | Underweight (<18.5 kg/m^2^) | – | – | Referent | – |
|  | Normal range (18.5-24.9 kg/m^2^) | – | – | -0.00 (-0.03 to 0.02) | 0.787 |
|  | Overweight (25-29.9 kg/m^2^) | – | – | 0.02 (-0.01 to 0.04) | 0.176 |
|  | Obese (≥30-kg/m^2^) | – | – | 0.02 (-0.01 to 0.05) | 0.189 |

Model 1, prevalent hypertension population (N=934) excludes participants with missing visit or experiential quality data. Model 2 consists of the same population as model 1 except the four participants with missing body mass index data were removed. *Age in years, adults aged ≥40 years. BMI, body mass index; CI, confidence interval; N, number.
