# Supplementary material for: Accessibility and quality of care for adults with hypertension in rural Burkina Faso: results from a cross-sectional household survey
Source: PLOS Glob Public Health. 2025 Apr 2;5(4):e0003161. doi: 10.1371/journal.pgph.0003161 (PMC11964235; doi:10.1371/journal.pgph.0003161)
Supplement: S6 Table — Model 2 excludes one participant with missing BMI data. *Age in years, adults aged ≥40 years. †27 participants dropped from analysis. BMI, body mass index; CI, confidence interval; N, number; POR, prevalence odds ratio. (DOCX) [file pgph.0003161.s009.docx]

**S6 Table.** **Multivariable regression to determine the association between participant characteristics and experience of care in the prevalent hypertension population who attended a healthcare facility in the last three months.**

| **Parameter** | **Group** | **Model 1 (N=250)** | | | | | |
| --- | --- | --- | --- | --- | --- | --- | --- |
|  |  | **Ease of following instructions** | | **Clarity of communication** | | **Involvement in treatment decisions** | |
|  |  | **POR (95% CI)** | **P value** | **POR (95% CI)** | **P value** | **POR (95% CI)** | **P value** |
| Gender | Male | Referent | – | Referent | – | Referent | – |
|  | Female | 0.69 (0.30 to 1.60) | 0.392 | 1.10 (0.34 to 3.60) | 0.871 | 0.81 (0.44 to 1.48) | 0.489 |
| Age* | – | 0.98 (0.94 to 1.02) | 0.239 | 1.03 (0.98 to 1.08) | 0.312 | 1.02 (1.00 to 1.05) | 0.095 |
| Education level | No formal education | Referent | – | Referent | – | Referent | – |
|  | Any education | 0.98 (0.34 to 2.79) | 0.965 | 0.98 (0.19 to 4.94) | 0.977 | 0.61 (0.28 to 1.32) | 0.209 |
| Marital status | Single/divorced/ widowed | Referent | – | Referent | – | Referent | – |
|  | Married/cohabiting | 1.11 (0.39 to 3.17) | 0.847 | 2.44 (0.59 to 10.19) | 0.221 | 0.93 (0.47 to 1.85) | 0.842 |
| Wealth quintile | 1 | Referent | – | Referent | – | Referent | – |
|  | 2 | 0.74 (0.15 to 3.63) | 0.708 | 0.33 (0.06 to 1.82) | 0.202 | 0.70 (0.25 to 1.95) | 0.496 |
|  | 3 | 1.72 (0.48 to 6.18) | 0.404 | 0.23 (0.04 to 1.28) | 0.094 | 1.25 (0.51 to 3.06) | 0.631 |
|  | 4 | 0.81 (0.21 to 3.22) | 0.770 | 0.54 (0.14 to 2.06) | 0.369 | 1.47 (0.61 to 3.51) | 0.389 |
|  | 5 | 0.98 (0.26 to 3.67) | 0.973 | **0.08 (0.01 to 0.71)** | **0.024** | 1.35 (0.57 to 3.18) | 0.492 |
| **Parameter** | **Group** | **Trust in skills and abilities of healthcare worker** | | **Opinion of medical providers knowledge and skills** | | **Borrowed or sold anything to pay for healthcare** | |
|  |  | **POR (95% CI)** | **P value** | **POR (95% CI)** | **P value** | **POR (95% CI)** | **P value** |
| Gender | Male | Referent | – | Referent | – | Referent | – |
|  | Female | 0.95 (0.43 to 2.09) | 0.904 | 0.77 (0.43 to 1.38) | 0.383 | 1.12 (0.47 to 2.69) | 0.799 |
| Age* | – | 1.00 (0.96 to 1.03) | 0.932 | 1.02 (1.00 to 1.05) | 0.059 | 1.01 (0.97 to 1.05) | 0.640 |
| Education level | No formal education | Referent | – | Referent | – | Referent | – |
|  | Any education | 0.39 (0.11 to 1.39) | 0.145 | 0.72 (0.35 to 1.49) | 0.377 | 0.82 (0.25 to 2.63) | 0.735 |
| Marital status | Single/divorced/ widowed | Referent | – | Referent | – | Referent | – |
|  | Married/cohabiting | 0.83 (0.34 to 2.01) | 0.681 | 1.52 (0.78 to 2.97) | 0.217 | 0.80 (0.31 to 2.04) | 0.633 |
| Wealth quintile | 1 | Referent | – | Referent | – | Referent | – |
|  | 2 | 0.70 (0.20 to 2.42) | 0.570 | 1.23 (0.48 to 3.20) | 0.667 | 0.95 (0.29 to 3.13) | 0.926 |
|  | 3 | 0.99 (0.34 to 2.89) | 0.982 | 1.28 (0.54 to 3.06) | 0.572 | 0.60 (0.18 to 1.98) | 0.402 |
|  | 4 | 0.80 (0.27 to 2.36) | 0.687 | 0.96 (0.41 to 2.25) | 0.931 | 0.52 (0.16 to 1.70) | 0.282 |
|  | 5 | 0.61 (0.20 to 1.84) | 0.379 | 1.09 (0.47 to 2.49) | 0.844 | 0.51 (0.16 to 1.61) | 0.252 |
| **Parameter** | **Group** | **Model 2 (N=249)** | | | | | |
|  |  | **Ease of following instructions** | | **Clarity of communication^†^** | | **Involvement in treatment decisions** | |
|  |  | **POR (95% CI)** | **P value** | **POR (95% CI)** | **P value** | **POR (95% CI)** | **P value** |
| Gender | Male | Referent | – | Referent | – | Referent | – |
|  | Female | 0.88 (0.37 to 2.11) | 0.778 | 1.08 (0.32 to 3.62) | 0.901 | 0.89 (0.48 to 1.67) | 0.725 |
| Age* | – | 0.97 (0.93 to 1.01) | 0.152 | 1.03 (0.98 to 1.09) | 0.237 | 1.02 (1.00 to 1.05) | 0.097 |
| Education level | No formal education | Referent | – | Referent | – | Referent | – |
|  | Any education | 0.82 (0.27 to 2.53) | 0.731 | 1.01 (0.20 to 5.19) | 0.994 | 0.67 (0.30 to 1.47) | 0.316 |
| Marital status | Single/divorced/ widowed | Referent | – | Referent | – | Referent | – |
|  | Married/cohabiting | 1.21 (0.41 to 3.58) | 0.725 | 2.42 (0.57 to 10.38) | 0.233 | 0.97 (0.48 to 1.95) | 0.932 |
| Wealth quintile | 1 | Referent | – | Referent | – | Referent | – |
|  | 2 | 1.01 (0.20 to 5.15) | 0.991 | 0.26 (0.04 to 1.52) | 0.134 | 0.68 (0.24 to 1.95) | 0.475 |
|  | 3 | 2.11 (0.56 to 8.00) | 0.270 | 0.18 (0.03 to 1.07) | 0.060 | 1.18 (0.47 to 2.98) | 0.723 |
|  | 4 | 1.10 (0.27 to 4.50) | 0.894 | 0.45 (0.11 to 1.80) | 0.259 | 1.47 (0.60 to 3.58) | 0.398 |
|  | 5 | 1.67 (0.43 to 6.51) | 0.461 | **0.08 (0.01 to 0.77)** | **0.028** | 1.53 (0.62 to 3.76) | 0.356 |
| BMI | Underweight (<18.5 kg/m^2^) | Referent | – | Referent | – | Referent | – |
|  | Normal range (18.5-25 kg/m^2^) | 0.49 (0.16 to 1.48) | 0.208 | 3.33 (0.38 to 29.1) | 0.277 | 0.92 (0.39 to 2.17) | 0.846 |
|  | Overweight (25-29.9 kg/m^2^) | 0.27 (0.07 to 1.08) | 0.064 | 6.53 (0.63 to 67.9) | 0.116 | 1.70 (0.64 to 4.52) | 0.285 |
|  | Obese (≥30-kg/m^2^) | **0.10 (0.01 to 1.00)** | **0.050** | 1.00 (empty) | – | 0.40 (0.11 to 1.51) | 0.178 |
| **Parameter** | **Group** | **Trust in skills and abilities of healthcare worker** | | **Opinion of medical providers knowledge and skills** | | **Borrowed or sold anything to pay for healthcare** | |
|  |  | **POR (95% CI)** | **P value** | **POR (95% CI)** | **P value** | **POR (95% CI)** | **P value** |
| Gender | Male | Referent | – | Referent | – | Referent | – |
|  | Female | 01.00 (0.45 to 2.24) | 0.995 | 0.78 (0.43 to 1.41) | 0.406 | 1.12 (0.45 to 2.78) | 0.804 |
| Age* | – | 1.00 (0.96 to 1.03) | 0.901 | **1.03 (1.00 to 1.06)** | **0.032** | 1.01 (0.97 to 1.05) | 0.605 |
| Education level | No formal education | Referent | – | Referent | – | Referent | – |
|  | Any education | 0.42 (0.12 to 1.54) | 0.192 | 0.69 (0.33 to 1.44) | 0.320 | 0.78 (0.24 to 2.51) | 0.674 |
| Marital status | Single/divorced/ widowed | Referent | – | Referent | – | Referent | – |
|  | Married/cohabiting | 0.87 (0.35 to 2.12) | 0.753 | 1.53 (0.78 to 3.01) | 0.220 | 0.73 (0.28 to 1.91) | 0.523 |
| Wealth quintile | 1 | Referent | – | Referent | – | Referent | – |
|  | 2 | 0.68 (0.19 to 2.45) | 0.558 | 1.11 (0.42 to 2.94) | 0.835 | 0.90 (0.26 to 3.10) | 0.866 |
|  | 3 | 0.91 (0.30 to 2.76) | 0.869 | 1.10 (0.45 to 2.68) | 0.838 | 0.61 (0.18 to 2.09) | 0.433 |
|  | 4 | 0.81 (0.27 to 2.48) | 0.714 | 0.89 (0.38 to 2.12) | 0.797 | 0.46 (0.14 to 1.53) | 0.205 |
|  | 5 | 0.65 (0.20 to 2.10) | 0.473 | 1.05 (0.44 to 2.52) | 0.906 | 0.53 (0.16 to 1.77) | 0.303 |
| BMI | Underweight (<18.5 kg/m^2^) | Referent | – | Referent | – | Referent | – |
|  | Normal range (18.5-25 kg/m^2^) | 0.72 (0.25 to 2.11) | 0.556 | 1.51 (0.65 to 3.52) | 0.334 | 3.75 (0.80 to 17.69) | 0.095 |
|  | Overweight (25-29.9 kg/m^2^) | 1.41 (0.44 to 4.55) | 0.567 | 2.19 (0.83 to 5.76) | 0.111 | 2.35 (0.41 to 13.43) | 0.336 |
|  | Obese (≥30-kg/m^2^) | 0.43 (0.07 to 2.66) | 0.364 | 1.28 (0.40 to 4.13) | 0.681 | 1.72 (0.20 to 14.97) | 0.623 |

Model 2 excludes one participant with missing BMI data. *Age in years, adults aged ≥40 years. ^†^27 observations were dropped from the analysis due to model fit. BMI, body mass index; CI, confidence interval; N, number; POR, prevalence odds ratio.
