# Supplementary material for: Accessibility and quality of care for adults with hypertension in rural Burkina Faso: results from a cross-sectional household survey
Source: PLOS Glob Public Health. 2025 Apr 2;5(4):e0003161. doi: 10.1371/journal.pgph.0003161 (PMC11964235; doi:10.1371/journal.pgph.0003161)
Supplement: S7 Table — Model 2 excludes one participant with missing BMI data. *Age in years, adults aged ≥40 years. BMI, body mass index; CI, confidence interval; N, number. (DOCX) [file pgph.0003161.s010.docx]

**S7 Table. Multivariable regression to determine the association between participant characteristics and shared understanding and decision making (SUDM) in participants with hypertension who attended a healthcare facility in the last three months.**

|  | | **Model 1 (N=250)** | | **Model 2 (N=249)** | |
| --- | --- | --- | --- | --- | --- |
| **Parameter** | **Group** | **Multivariable analysis coefficient (95% CI)** | **P value** | **Multivariable analysis coefficient (95% CI)** | **P value** |
| Gender | Male | Referent | – | Referent | – |
|  | Female | -0.01 (-0.04 to 0.02) | 0.535 | -0.01 (-0.04 to 0.02) | 0.595 |
| Age* | – | 0.001 (-0.00 to 0.00) | 0.106 | 0.00 (-0.00 to 0.00) | 0.081 |
| Education level | No formal education | Referent | – | Referent | – |
|  | Any education | -0.03 (-0.07 to 0.01) | 0.179 | -0.03 (-0.07 to 0.02) | 0.230 |
| Marital status | Single/divorced/ widowed | Referent | – | Referent | – |
|  | Married/cohabiting | 0.01 (-0.02 to 0.05) | 0.482 | 0.01 (-0.02 to 0.05) | 0.468 |
| Wealth quintile | 1 | Referent | – | Referent | – |
|  | 2 | -0.02 (-0.07 to 0.04) | 0.535 | -0.02 (-0.08 to 0.03) | 0.436 |
|  | 3 | 0.00 (-0.05 to 0.05) | 0.900 | -0.00 (-0.05 to 0.05) | 0.932 |
|  | 4 | -0.00 (-0.05 to 0.05) | 0.968 | -0.00 (-0.05 to 0.04) | 0.852 |
|  | 5 | 0.00 (-0.05 to 0.05) | 0.943 | 0.00 (-0.05 to 0.05) | 0.956 |
| BMI | Underweight (<18.5 kg/m^2^) | – | – | Referent | – |
|  | Normal range (18.5-24.9 kg/m^2^) | – | – | 0.01 (-0.03 to 0.06) | 0.579 |
|  | Overweight (25-29.9 kg/m^2^) | – | – | 0.04 (-0.01 to 0.09) | 0.148 |
|  | Obese (≥30 kg/m^2^) | – | – | -0.00 (-0.07 to 0.06) | 0.897 |

Model 2 excludes one participant with missing BMI data. *Age in years, adults aged ≥40 years. BMI, body mass index; CI, confidence interval; N, number.
