# Supplementary material for: Accessibility and quality of care for adults with hypertension in rural Burkina Faso: results from a cross-sectional household survey
Source: PLOS Glob Public Health. 2025 Apr 2;5(4):e0003161. doi: 10.1371/journal.pgph.0003161 (PMC11964235; doi:10.1371/journal.pgph.0003161)
Supplement: S8 Table — *Model 1 and model 2 exclude one participant with missing health system quality outcome data. †Age in years, adults aged ≥40 years. **27 participants dropped from reported met need analysis. BMI, body mass index; CI, confidence interval; N, number. POR, prevalence odds ratio. (DOCX) [file pgph.0003161.s011.docx]

**S8 Table.** **Multivariable regression to show the association between health system quality outcomes and participant sociodemographic characteristics in participants with hypertension that accessed care in the last three months.**

|  | | **Model 1 (N=249)*** | | | | | |
| --- | --- | --- | --- | --- | --- | --- | --- |
|  |  | **Reported met need** | | **Trust and confidence in health care system** | | **Overall view of the health care system** | |
| **Parameter** | **Group** | **POR (95% CI)** | **P value** | **POR (95% CI)** | **P value** | **POR (95% CI)** | **P value** |
| Gender | Male | Referent | – | Referent | – | Referent | – |
|  | Female | 0.82 (0.29 to 2.27) | 0.701 | 0.73 (0.41 to 1.32) | 0.296 | 0.83 (0.46 to 1.52) | 0.551 |
| Age^†^ | – | 0.97 (0.93 to 1.01) | 0.178 | 0.98 (0.96 to 1.01) | 0.145 | 0.98 (0.95 to 1.01) | 0.139 |
| Education level | No formal education | Referent | – | Referent | – | Referent | – |
|  | Any education | 0.42 (0.09 to 2.04) | 0.283 | 0.77 (0.37 to 1.61) | 0.486 | 0.70 (0.33 to 1.46) | 0.337 |
| Marital status | Single/ divorced/ widowed | Referent | – | Referent | – | Referent | – |
|  | Married/ cohabiting | 0.51 (0.17 to 1.57) | 0.240 | 1.59 (0.79 to 3.20) | 0.193 | 0.83 (0.42 to 1.65) | 0.594 |
| Wealth quintile | 1 | Referent | – | Referent | – | Referent | – |
|  | 2 | 0.53 (0.12 to 2.36) | 0.404 | 0.75 (0.28 to 1.99) | 0.561 | 0.94 (0.34 to 2.61) | 0.903 |
|  | 3 | 0.56 (0.15 to 2.10) | 0.393 | 0.75 (0.31 to 1.83) | 0.525 | 0.56 (0.22 to 1.40) | 0.217 |
|  | 4 | 0.39 (0.10 to 1.55) | 0.181 | 0.65 (0.27 to 1.57) | 0.341 | 1.04 (0.41 to 2.60) | 0.935 |
|  | 5 | 0.46 (0.12 to 1.76) | 0.258 | 0.68 (0.29 to 1.60) | 0.374 | 0.54 (0.23 to 1.29) | 0.167 |
|  | | **Model 2 (N=248)** | | | | | |
|  |  | **Reported met need**** | | **Trust and confidence in health care system** | | **Overall view of the health care system** | |
| **Parameter** | **Group** | **POR (95% CI)** | **P value** | **POR (95% CI)** | **P value** | **POR (95% CI)** | **P value** |
| Gender | Male | Referent | – | Referent | – | Referent | – |
|  | Female | 0.87 (0.30 to 2.51) | 0.791 | 0.83 (0.45 to 1.51) | 0.541 | 0.85 (0.56 to 1.59) | 0.612 |
| Age^†^ | – | 0.97 (0.93 to 1.02) | 0.230 | 0.98 (0.95 to 1.00) | 0.078 | 0.98 (0.95 to 1.01) | 0.135 |
| Education level | No formal education | Referent | – | Referent | – | Referent | – |
|  | Any education | 0.44 (0.09 to 2.14) | 0.311 | 0.83 (0.39 to 1.76) | 0.623 | 0.74 (0.35 to 1.57) | 0.431 |
| Marital status | Single/ divorced/ widowed | Referent | – | Referent | – | Referent | – |
|  | Married/ cohabiting | 0.49 (0.15 to 1.57) | 0.230 | 1.64 (0.81 to 3.34) | 0.171 | 0.80 (0.40 to 1.61) | 0.539 |
| Wealth quintile | 1 | Referent | – | Referent | – | Referent | – |
|  | 2 | 0.47 (0.10 to 2.17) | 0.330 | 0.87 (0.32 to 2.37) | 0.782 | 0.93 (0.33 to 2.66) | 0.894 |
|  | 3 | 0.48 (0.12 to 1.88) | 0.292 | 0.88 (0.35 to 2.21) | 0.792 | 0.59 (0.23 to 1.52) | 0.274 |
|  | 4 | 0.33 (0.08 to 1.35) | 0.121 | 0.71 (0.29 to 1.74) | 0.452 | 0.98 (0.38 to 2.50) | 0.964 |
|  | 5 | 0.50 (0.13 to 1.98) | 0.322 | 0.87 (0.35 to 2.14) | 0.757 | 0.57 (0.23 to 1.42) | 0.227 |
| BMI | Underweight (<18.5 kg/m^2^) | Referent | – | Referent | – | Referent | – |
|  | Normal range (18.5-25 kg/m^2^) | 4.26 (0.51 to 35.6) | 0.181 | 0.77 (0.33 to 1.81) | 0.549 | 1.59 (0.68 to 3.72) | 0.282 |
|  | Overweight (25-30 kg/m^2^) | 6.46 (0.71 to 59.1) | 0.099 | 0.65 (0.25 to 1.73) | 0.390 | 1.35 (0.51 to 3.58) | 0.542 |
|  | Obese (≥30-kg/m^2^) | 1.00 (empty) | – | 0.29 (0.08 to 1.05) | 0.060 | 0.93 (0.29 to 2.98) | 0.902 |

*Model 1 and model 2 exclude one participant with missing health system quality outcome data. ^†^Age in years, adults aged ≥40 years. **27 observations were dropped from reported met need analysis due to model fit. BMI, body mass index; CI, confidence interval; N, number. POR, prevalence odds ratio.
