# Supplementary material for: Accessibility and quality of care for adults with hypertension in rural Burkina Faso: results from a cross-sectional household survey
Source: PLOS Glob Public Health. 2025 Apr 2;5(4):e0003161. doi: 10.1371/journal.pgph.0003161 (PMC11964235; doi:10.1371/journal.pgph.0003161)
Supplement: S9 Table — Model 2 Hypertension population includes those identified as hypertensive when examined by a study investigator and/or those who have previously been told they have hypertension and/or those who are currently taking treatment for hypertension, excludes participants with missing BMI data (N=1004). The denominator population at each step of the cascade includes the participants that achieved the previous step. For example, the denominator population for the analysis of the association between sociodemographic characteristics and diagnosed vs not, are the participants that were screened for hypertension in the previous step of the cascade. Prevalent hypertension population (N=1002), screened (N=623), diagnosed (N=428), treated (N=149), controlled disease (N=68). *Age in years, adults aged ≥40 years. BMI, body mass index; CI, confidence interval; N, number; POR, prevalence odds ratio. (DOCX) [file pgph.0003161.s012.docx]

**S9 Table. Multivariable association between sociodemographic characteristics and likelihood of progressing through the care cascade (model 2).**

|  |  | **Screened vs not** | | **Diagnosed vs not** | | **Treated vs not** | | **Controlled vs not** | |
| --- | --- | --- | --- | --- | --- | --- | --- | --- | --- |
| **Parameter** | **Group** | **POR (95% CI)** | **P value** | **POR (95% CI)** | **P value** | **POR (95% CI)** | **P value** | **POR (95% CI)** | **P value** |
| Gender | Male | Referent | – | Referent | – | Referent | – | Referent | – |
|  | Female | **1.66 (1.21 to 2.28)** | **0.002** | **1.51 (1.02 to 2.25)** | **0.041** | 1.57 (0.96 to 2.56) | 0.073 | 1.61 (0.69 to 3.78) | 0.273 |
| Age* |  | 1.01 (1.00 to 1.03) | 0.095 | 1.00 (0.99 to 1.02) | 0.615 | **1.04 (1.01 to 1.06)** | **0.001** | 0.99 (0.95 to 1.02) | 0.458 |
| Education level | No formal education | Referent | – | Referent | – | Referent | – | Referent | – |
|  | Any education | **1.68 (1.08 to 2.60)** | **0.020** | 0.86 (0.54 to 1.35) | 0.511 | 1.02 (0.58 to 1.80) | 0.933 | 1.36 (0.53 to 3.46) | 0.520 |
| Marital status | Single/ divorced/ widowed | Referent | – | Referent | – | Referent | – | Referent | – |
|  | Married/ cohabiting | **1.62 (1.12 to 2.33)** | **0.010** | 0.99 (0.62 to 1.59) | 0.967 | 0.87 (0.51 to 1.50) | 0.622 | 0.77 (0.32 to 1.88) | 0.569 |
| Wealth quintile | 1 | Referent | – | Referent | – | Referent | – | Referent | – |
|  | 2 | 1.45 (0.94 to 2.24) | 0.090 | 1.35 (0.69 to 2.61) | 0.379 | 0.56 (0.24 to 1.31) | 0.185 | 1.76 (0.41 to 7.59) | 0.451 |
|  | 3 | **1.76 (1.15 to 2.71)** | **0.010** | 1.12 (0.60 to 2.10) | 0.718 | 0.45 (0.20 to 1.04) | 0.061 | 0.48 (0.11 to 1.99) | 0.311 |
|  | 4 | **2.71 (1.78 to 4.14)** | **<0.001** | 1.28 (0.71 to 2.31) | 0.412 | 0.80 (0.38 to 1.66) | 0.550 | 0.37 (0.11 to 1.25) | 0.110 |
|  | 5 | **5.77 (3.57 to 9.31)** | **<0.001** | 1.41 (0.78 to 2.54) | 0.252 | 1.06 (0.52 to 2.17) | 0.878 | 0.96 (0.30 to 3.00) | 0.940 |
| BMI | <18.5 kg/m^2^ | Referent | – | Referent | – | Referent | – | Referent | – |
|  | 18.5-24.9 kg/m^2^ | 1.36 (0.92 to 2.02) | 0.128 | 0.92 (0.52 to 1.63) | 0.771 | 1.63 (0.76 to 3.50) | 0.210 | 2.55 (0.64 to 10.22) | 0.185 |
|  | 25-29.9 kg/m^2^ | 1.54 (0.94 to 2.52) | 0.090 | 1.32 (0.67 to 2.62) | 0.422 | **2.65 (1.13 to 6.22)** | **0.026** | 1.89 (0.42 to 8.49) | 0.407 |
|  | ≥30-kg/m^2^ | **2.91 (1.41 to 6.03)** | **0.004** | 1.44 (0.64 to 3.21) | 0.375 | 2.44 (0.94 to 6.31) | 0.066 | 1.17 (0.23 to 5.91) | 0.850 |

Model 2 Hypertension population includes those identified as hypertensive when examined by a study investigator, those who have previously been told they have hypertension and those who are currently taking treatment for hypertension, excludes participants with missing BMI data (N=1004). The denominator population at each step of the cascade includes the participants that achieved the previous step. For example, the denominator population for the analysis of the association between sociodemographic characteristics and diagnosed vs not, are the participants that were screened for hypertension in the previous step of the cascade. Prevalent hypertension population (N=1002), screened (N=623), diagnosed (N=428), treated (N=149), controlled disease (N=68). *Age in years, adults aged ≥40 years. BMI, body mass index; CI, confidence interval; N, number; POR, prevalence odds ratio.
