# Supplementary material for: Accessibility and quality of care for adults with hypertension in rural Burkina Faso: results from a cross-sectional household survey
Source: PLOS Glob Public Health. 2025 Apr 2;5(4):e0003161. doi: 10.1371/journal.pgph.0003161 (PMC11964235; doi:10.1371/journal.pgph.0003161)
Supplement: S10 Table — *Overall population with hypertension excludes four participants with missing BMI data. †Age in years, adults aged ≥40 years. BMI, body mass index; CI, confidence interval; N, number; POR, prevalence odds ratio. (DOCX) [file pgph.0003161.s013.docx]

**S10 Table.** **Multivariable regression shows the association between odds of timely access to care (healthcare appointment within the previous three months vs not) and participant characteristics representative of equity (model 2).**

| **Model 2. Overall population with hypertension N=1000*, adjusted for Body Mass Index (BMI)** | | | |
| --- | --- | --- | --- |
|  |  |  |  |
| **Parameter** | **Group** | **POR** | **P value** |
|  |  | **95% CI** |  |
| Gender | Male | Referent | – |
|  | Female | 1.20 (0.86 to 1.66) | 0.278 |
| Age**^†^** | – | 1.01 (1.00 to 1.03) | 0.057 |
| Education level | No formal education | Referent | – |
|  | Any education | 0.97 (0.64 to 1.46) | 0.874 |
| Marital status | Single/divorced/ widowed | Referent | – |
|  | Married/cohabiting | 0.94 (0.64 to 1.37) | 0.737 |
| Wealth quintile | 1 | Referent | – |
|  | 2 | 1.10 (0.66 to 1.84) | 0.714 |
|  | 3 | **1.66 (1.02 to 2.69)** | **0.041** |
|  | 4 | **1.66 (1.04 to 2.65)** | **0.033** |
|  | 5 | **1.62 (1.00 to 2.61)** | **0.050** |
| BMI | Underweight (<18.5 kg/m^2^) | Referent | – |
|  | Normal range (18.5-24.9 kg/m^2^) | 0.92 (0.60 to 1.42) | 0.710 |
|  | Overweight (25-30 kg/m^2^) | 1.28 (0.76 to 2.15) | 0.358 |
|  | Obese (≥30-kg/m^2^) | 1.27 (0.67 to 2.39) | 0.465 |

*Overall population with hypertension excludes four participants with missing BMI data. ^†^Age in years, adults aged ≥40 years. BMI, body mass index; CI, confidence interval; N. number; POR, prevalence odds ratio.
