# Supplementary material for: Accessibility and quality of care for adults with hypertension in rural Burkina Faso: results from a cross-sectional household survey
Source: PLOS Glob Public Health. 2025 Apr 2;5(4):e0003161. doi: 10.1371/journal.pgph.0003161 (PMC11964235; doi:10.1371/journal.pgph.0003161)
Supplement: S11 Table — *Two participants excluded due to missing health system quality outcome data. †Age in years, adults aged ≥40 years. BMI, body mass index; CI, confidence interval; N, number; POR, prevalence odds ratio. (DOCX) [file pgph.0003161.s014.docx]

**S11 Table. Multivariable regression to show the association between health system quality outcomes and participant sociodemographic characteristics in participants with hypertension regardless of timeliness of access to care (model 2).**

|  | | **Model 2 (N=928)*** | | | | | |
| --- | --- | --- | --- | --- | --- | --- | --- |
|  |  | **Reported met need** | | **Trust and confidence in health care system** | | **Overall view of the health care system** | |
| **Parameter** | **Group** | **POR (95% CI)** | **P value** | **POR (95% CI)** | **P value** | **POR (95% CI)** | **P value** |
| Gender | Male | Referent | – | Referent | – | Referent | – |
|  | Female | 0.98 (0.73 to 1.32) | 0.916 | 0.77 (0.56 to 1.06) | 0.112 | 0.77 (0.57 to 1.04) | 0.091 |
| Age^†^ | – | 1.00 (0.99 to 1.01) | 0.869 | 0.99 (0.98 to 1.00) | 0.154 | 0.99 (0.98 to 1.01) | 0.284 |
| Education level | No formal education | Referent | – | Referent | – | Referent | – |
|  | Any education | 0.98 (0.68 to 1.42) | 0.912 | 0.97 (0.66 to 1.44) | 0.898 | 0.84 (0.58 to 1.23) | 0.367 |
| Marital status | Single/ divorced/ widowed | Referent | – | Referent | – | Referent | – |
|  | Married/ cohabiting | 0.99 (0.69 to 1.41) | 0.959 | 1.21 (0.82 to 1.78) | 0.347 | 0.85 (0.60 to 1.23) | 0.392 |
| Wealth quintile | 1 | Referent | – | Referent | – | Referent | – |
|  | 2 | 1.42 (0.90 to 2.25) | 0.131 | 0.97 (0.58 to 1.61) | 0.894 | 1.01 (0.63 to 1.61) | 0.971 |
|  | 3 | 1.53 (0.98 to 2.38) | 0.064 | 1.12 (0.69 to 1.83) | 0.651 | 1.07 (0.68 to 1.69) | 0.757 |
|  | 4 | 1.36 (0.88 to 2.09) | 0.163 | 1.55 (0.97 to 2.46) | 0.064 | 0.97 (0.63 to 1.49) | 0.874 |
|  | 5 | **1.94 (1.25 to 3.02)** | **0.003** | **1.62 (1.01 to 2.60)** | **0.046** | 0.94 (0.61 to 1.47) | 0.798 |
| BMI | Underweight (<18.5 kg/m^2^) | Referent | – | Referent | – | Referent | – |
|  | Normal range (18.5-25 kg/m^2^) | 0.98 (0.64 to 1.48) | 0.911 | 0.65 (0.42 to 1.00) | 0.052 | 0.94 (0.61 to 1.44) | 0.799 |
|  | Overweight (25-30 kg/m^2^) | 1.15 (0.70 to 1.88) | 0.584 | 0.60 (0.36 to 1.02) | 0.060 | 0.84 (0.51 to 1.40) | 0.512 |
|  | Obese (≥30-kg/m^2^) | 0.86 (0.47 to 1.59) | 0.638 | **0.52 (0.27 to 1.00)** | **0.049** | 0.73 (0.40 to 1.36) | 0.324 |

*Two participants excluded due to missing health system quality outcome data. ^†^Age in years, adults aged ≥40 years. BMI, body mass index; CI, confidence interval; N, number; POR, prevalence odds ratio.
