# Supplementary material for: Accessibility and quality of care for adults with hypertension in rural Burkina Faso: results from a cross-sectional household survey
Source: PLOS Glob Public Health. 2025 Apr 2;5(4):e0003161. doi: 10.1371/journal.pgph.0003161 (PMC11964235; doi:10.1371/journal.pgph.0003161)
Supplement: Attachment — Submitted filename: DBazira Peer Review -PLOS GLOBAL HEALTH-PGPH-D-24-00560.pdf [file pgph.0003161.s015.pdf]

**<u>Manuscript Review: Accessibility of care and experiential quality of care for adults with hypertension in rural Burkina Faso: results from a cross-sectional household survey</u> (Deus Bazira)**

Thank you for the opportunity to review this paper. I acknowledge the considerable efforts the authors have put into conducting this cross-sectional study for hypertension in rural Burkina Faso. The primary data collection process and obtaining ethical approval are appreciated, and the paper is clearly written, delivering key findings to the readers of PLOS Global Public Health. However, I have several concerns regarding the study's design and analysis methodologies that need to be addressed before the manuscript can be considered for publication.

1. The paper does not clearly define and quantify the term "wealth quintile." Please provide a clear definition and the methodology used to categorize the population into different wealth quintiles to ensure understanding of your findings.
2. Given that this is a cross-sectional study, it is more appropriate to report findings using Prevalence Ratio rather than Odds Ratio. The Odds Ratio is typically used for cohort studies. Please consider revision of your statistical analysis accordingly to better reflect the study design, or provide rationale why you chose Odds Ratio.
3. Hypertension is a chronic disease that generally requires longitudinal data to understand its progression and related factors comprehensively. A cross-sectional design may not be the most suitable for such a condition. A retrospective or prospective cohort study would provide more robust data. The lack of longitudinal data collection might significantly affect the study's validity. Please justify your choice of a cross-sectional design in the context of hypertension research or at a minimum amplify this weakness in your discussions and conclusion.
4. Patient-centered care (patient-centeredness) is best measured from both the supply (health system and provider) and patient perspectives. Drawing strong conclusions about quality of care and hence patient-centeredness in care from only the patient side, using data that is heavily reliant on ability of patients to recall their experience receiving and/or navigating care is overstretching it. This major weakness needs to be significantly acknowledged and hence the conclusions drawn from the study should be tapered. Relatedly, drawing conclusion about accessibility & quality of care based purely on reported patient experiences and recall without consideration of supply-side measures is the main weakness of this study and it should be highlighted when discussing and presenting major study findings.
5. The use of multivariable regression models, as described, may not be the best approach. Use of multivariate regression models, which consider multiple dependent variables simultaneously, providing a more accurate estimation, could be considered a better alternative. Did you consider this? Is it feasible for you to rebuild your models using multivariate regression techniques as an option?
6. Gender, education, and income are well-known confounders or effect modifiers that can significantly influence the estimated associations. It would enhance the study's robustness if you included an analysis of these potential confounders and effect modifiers.
7. The current figures in the manuscript are of low resolution. Please improve the quality and resolution of all figures.
8. The readability of the paper would greatly benefit from further English editing and polishing.
