# Supplementary material for: Accessibility and quality of care for adults with hypertension in rural Burkina Faso: results from a cross-sectional household survey
Source: PLOS Glob Public Health. 2025 Apr 2;5(4):e0003161. doi: 10.1371/journal.pgph.0003161 (PMC11964235; doi:10.1371/journal.pgph.0003161)
Supplement: Attachment — Submitted filename: Response to reviewers.docx [file pgph.0003161.s018.docx]

**Response to reviewer’s comments**

Dear reviewers,

I would like to thank the editor and reviewers for reviewing this paper. Please find a detailed response to each comment below.

Kind regards

Stephanie Lacey

**Reviewer 1**

This is an excellent manuscript covering an important topic for improving the services provided to patients with hypertension in Burkina Faso. The manuscript is well written and statistical analyses are robustly done. There are few very minor areas that I would like to suggest to authors to look at as follows:

Introduction

Line 105: Write WHO in its long form and the abbreviation in brackets.

Line 115: Edit “IOMs” by adding an apostrophe between M and s so that it will be as follows: IOM's.

Methodology

Line 147 (categorised in quintiles using the principal component method of Filmer and Pritchett. We): I suggest to the authors to add a reference for Filmer and Pritchett in order to assist a reader(s) to get more details as may be needed.

Line 166 (investigators was <140/90mmHg, as defined by the World Health Organisations Package of): I suggest to the authors to replace “World Health Organisations” with the WHO's.

Discussion

Line 421 (We found some inequity in experiential quality. Mostly related to wealth, with those people): I suggest to the authors to EDIT the sentence between the two words (…..quality. Mostly…..) by removing full stop and the word "Mostly" to be with a small "m".

Conclusion

Line 473 (We found that quality of care for hypertension as determined by the Institute of Medicine): I suggest to the authors to replace “Institute of Medicine” with the following: IOM (currently National Academy of Medicine).

**Reply:** Thank you for your suggestions to improve the readability of our paper. Your suggested changes have been incorporated into the manuscript. Please see track-changes throughout the manuscript. Some examples include, tracked changes page 5, lines 107-108; page 8, line 172; and page 28, lines 512-513:

“*The World Health Organization (WHO)* Stepwise approach to Surveillance (STEPS) survey conducted in 2013…”

“*WHO’s* Package of Essential Non-communicable disease (NCD) interventions (*WHO* PEN)”

“We found that quality of care for people with hypertension as determined by the *IoM (currently National Academy of Medicine)* domains…”

**Reviewer 2**

Thank you for the opportunity to review this paper. I acknowledge the considerable efforts the authors have put into conducting this cross-sectional study for hypertension in rural Burkina Faso. The primary data collection process and obtaining ethical approval are appreciated, and the paper is clearly written, delivering key findings to the readers of PLOS Global Public Health. However, I have several concerns regarding the study's design and analysis methodologies that need to be addressed before the manuscript can be considered for publication.

**Reply:** Thank you for reviewing our paper and making recommendations to enhance the clarity and quality of this publication. We are very happy to hear that you appreciate our work.

**Point 1:** The paper does not clearly define and quantify the term "wealth quintile." Please provide a clear definition and the methodology used to categorize the population into different wealth quintiles to ensure understanding of your findings.

**Reply:** Dividing a population into wealth quintiles (with one quintile representing a fifth [20%] of the population) is one way of understanding relative poverty in a population. Wealth quintiles can also be used to understand how equitable the distribution of other indicators is in a population according to wealth e.g., timeliness access to healthcare. Creation of wealth quintiles from household assets is commonly used in low- or middle-income countries where household income data may not be readily available. The approach we used to create wealth quintiles using data on household assets employs a Principal Component Analysis approach and was developed by Filmer and Pritchett as referenced in the manuscript. Principal Component Analysis assigns a factor weight to each asset or household characteristic so that a wealth index score can be calculated for each participant. The participant wealth index scores are then ranked and divided into five equal groups (quintiles), with the first quintile representing the poorest households and the fifth quintile representing the wealthiest households. This has now been explained in closer detail in the manuscript.

Please see track changes page 7, lines 149-154, and S1 Table in the supplementary information file.

In the text:

*“In short, study participants were asked to complete survey questions related to household assets deemed indicative of wealth. Each asset was assigned a factor weight and a wealth index score was calculated for each participant. Participant scores were ranked and divided into five equal groups (quintiles) (see S1 Table for further information on wealth quintile generation and household asset survey questions used) [24]”.*

In the supplementary information (page 1, in relation to S1 Table):

*“Dividing a population into wealth quintiles (with one quintile representing a fifth [20%] of the population) is one way of understanding relative poverty in a population. Wealth quintiles can also be used to understand how equitable the distribution of other indicators is in a population according to wealth e.g., timeliness access to healthcare. The Filmer and Pritchett method for calculating wealth quintiles is a commonly used approach in low- or middle-income countries where household income data may not be readily available [1]. This approach determines household wealth based on asset ownership and household characteristics. Participants are asked to complete a survey containing variables deemed relevant in that setting to be indicative of wealth e.g., owns a fridge or TV. Principal Component Analysis is then used to assign a factor weight to each asset or household characteristic so that a wealth index score can be calculated for each participant. The participant wealth index scores are then ranked and divided into five equal groups (quintiles), with the first quintile representing the poorest households and fifth quintile representing the wealthiest households [2, 3].”*

**Point 2:** Given that this is a cross-sectional study, it is more appropriate to report findings using Prevalence Ratio rather than Odds Ratio. The Odds Ratio is typically used for cohort studies. Please consider revision of your statistical analysis accordingly to better reflect the study design, or provide rationale why you chose Odds Ratio.

**Reply:** We have carefully considered your comment and discussed with the epidemiologists on the author team. Whilst the prevalence ratio is a suitable statistical method that could have been used in our analysis of cross-sectional household data, we chose to construct binary logistic regression models which are widely used to effectively adjust for multiple confounders in the analysis of survey data, including cross-sectional study data. We present odds ratios in our paper as they are the natural measure of association from logistic regression models. They are also widely understood by readers and used in other previously published studies from this dataset (<https://doi.org/10.1016/S2666-7568(24)00096-5>) and from other datasets (<https://doi.org/10.1016/j.eclinm.2023.102226>). We also have used odds ratio as we wish to maintain consistency in reporting across publications using this dataset. The odds ratio also offers a reasonable approximation to prevalence ratios when the outcome of interest is rare or very common. Therefore, we do not anticipate that changing the model to present prevalence ratios would have a meaningful impact on the study findings.

**Point 3:** Hypertension is a chronic disease that generally requires longitudinal data to understand its progression and related factors comprehensively. A cross-sectional design may not be the most suitable for such a condition. A retrospective or prospective cohort study would provide more robust data. The lack of longitudinal data collection might significantly affect the study's validity. Please justify your choice of a cross-sectional design in the context of hypertension research or at a minimum amplify this weakness in your discussions and conclusion.

**Reply:** We agree on the greater value of longitudinal data. This study leveraged existing cross-sectional data on which we conducted a secondary analysis. The cross-sectional study design offered a number of benefits appropriate for the local context. Cross-sectional studies are still valuable to emerge findings or associations at a single point in time, even if attribution is not possible. Cross-sectional studies also still permit comprehensive data collection of various factors simultaneously, allowing a broad understanding of the quality of care for patients with hypertension and the identification of associations between quality-of-care indicators and patient demographic factors, informing hypothesis generation for future research. Cross-sectional studies provide a snapshot of the current situation and are useful in settings with little prior knowledge, providing policymakers and healthcare professionals with immediate insights into areas that require improvement. Cross-sectional studies have been used in other low-and-middle income settings to understand the state of care for hypertension and diabetes (<https://doi.org/10.1016/s0140-6736(19)30955-9> and <https://doi.org/10.1371%2Fjournal.pmed.1002751>). Similarly, the World Health Organization’s Stepwise approach to Surveillance cross-sectional studies have frequently been used to estimate the prevalence of hypertension and factors associated with hypertension (<https://doi.org/10.1186/s12889-016-3926-8>).

We have elaborated on this in the limitations section. Please see track changes page 27, lines 484-491.

*“We did not have longitudinal data to fully understand aspects of care quality that might require a longer time horizon to capture, such as disease monitoring and patient follow-up. Understanding the experiential quality process and care outcome measures was limited to the patient’s most recent interaction with the health service. Whilst cross-sectional study designs can identify associations between participant characteristics and quality of care measures, they cannot demonstrate causality. However, cross-sectional studies are frequently used to understand the current position of chronic disease management [10, 28].”*

**Point 4:** Patient-centered care (patient-centeredness) is best measured from both the supply (health system and provider) and patient perspectives. Drawing strong conclusions about quality of care and hence patient-centeredness in care from only the patient side, using data that is heavily reliant on ability of patients to recall their experience receiving and/or navigating care is overstretching it. This major weakness needs to be significantly acknowledged and hence the conclusions drawn from the study should be tapered. Relatedly, drawing conclusion about accessibility & quality of care based purely on reported patient experiences and recall without consideration of supply-side measures is the main weakness of this study and it should be highlighted when discussing and presenting major study findings.

**Reply:** We appreciate your observations regarding measuring quality of care and patient-centred care and the limitations of relying solely on patient-reported experiences. However, our assessment of care quality includes patient-centredness as well as three other domains of the six Institute of Medicine quality of care framework, including questions based on the WHO STEPs survey which is widely utilised to assess effectiveness of care, through the construction of care cascades which uses both self-reported and measured outcome variables, as described in our methods section. The IoM domain of patient-centredness, is by definition self-reported as this captures the patient experiences of care. It is also a widely accepted approach (e.g. DOI: 10.1093/intqhc/mzad027). We acknowledge this is limited by recall bias in the limitations section. We realise that the title of the manuscript “experiential quality” may have been misleading and have therefore amended to reflect the broader assessment of care quality.

Please see track changes page 1, lines 1-2; and page 27, lines 501-509.

*“Accessibility and quality of care for adults with hypertension in rural Burkina Faso: results from a cross-sectional household survey”.*

*“We note that we do not present any formal assessment of the health system in this study and are therefore unable to present to the IoM domain of safety. When considering effectiveness, we also have not done a formal assessment of process measures such as provider competency or facility readiness to provide care (such as availability of medicines, affordability of care, workforce training, leadership, governance, record management, continuity of care); rather we have used clinical outcomes to assess if the health system is delivering effective outcomes. We also did not ask healthcare workers about their respect for patient values and preferences, as the outcome of importance is patients’ experience of the health system*”.

**Point 5:** The use of multivariable regression models, as described, may not be the best approach. Use of multivariate regression models, which consider multiple dependent variables simultaneously, providing a more accurate estimation, could be considered a better alternative. Did you consider this? Is it feasible for you to rebuild your models using multivariate regression techniques as an option?

**Reply:** The primary objectives of our study were to describe the population prevalence of hypertension and to assess the quality of care in terms of effectiveness, timeliness, patient-centeredness, and equitability. These objectives focus on understanding how individual predictors, e.g., age, gender, and wealth, influence specific outcomes related to experiential care quality so that we can provide specific, actionable insights for policymakers and healthcare professionals. While we acknowledge the value of analysing multiple dependent variables simultaneously, we believe multivariable regression is the most appropriate and feasible method for our study objectives. We also acknowledge the ease of reader-interpretation of the results from the methods that we had used, which is why we selected them in the first instance. To re-analyse the data as suggested would require resources beyond that which we can allocate to the project and, we believe, do not enhance the interpretation of the findings for a general reader. We are confident that the multivariable regression approach, commonly used to explore study objectives in work similar to ours, will be familiar to readers in this field (<https://doi.org/10.1371/journal.pmed.1004140>).

**Point 6:** Gender, education, and income are well-known confounders or effect modifiers that can significantly influence the estimated associations. It would enhance the study's robustness if you included an analysis of these potential confounders and effect modifiers.

**Reply:** We agree that gender, education, and income are important factors that can influence these associations. Whilst we do not have information on income (as described above, we use wealth quintiles), we do include sex, education, and wealth as variables indicative of equity.

**Point 7:** The current figures in the manuscript are of low resolution. Please improve the quality and resolution of all figures.

**Reply:** The resolution of the figures has been reviewed in the Preflight Analysis and Conversion Engine (PACE) digital diagnostic tool to ensure they meet the requirements as per PLOS Global Health submission guidelines. If any further issues with the resolution we can liaise with the production team.

**Point 8:** The readability of the paper would greatly benefit from further English editing and polishing.

**Reply:** We have now reread the text several times and reviewed grammatical accuracy, punctuation and spelling, and consistency of terminology and style throughout the manuscript. Please see other smaller amendments made as tracked changes throughout the manuscript.
