# Supplementary material for: Accessibility and quality of care for adults with hypertension in rural Burkina Faso: results from a cross-sectional household survey
Source: PLOS Glob Public Health. 2025 Apr 2;5(4):e0003161. doi: 10.1371/journal.pgph.0003161 (PMC11964235; doi:10.1371/journal.pgph.0003161)
Supplement: Attachment — Submitted filename: Review-PGPH-24-00560-R1-10-29-2024.docx [file pgph.0003161.s017.docx]

Thank you for your detailed response to my previous feedback, I appreciate the authors’ efforts. Most of my concerns were fully addressed, but I recommend the following:

1: Please revise the term “odds ratios” to “prevalence odds ratios.” While the calculation is the same for both, “prevalence odds ratios” is more accurate for a cross-sectional study design, and will help avoid confusion for readers with epidemiological expertise.

2. Since you have chosen the use of **multivariable regression models** in this context, your rebuttal notwithstanding, I recommend you include a statement that acknowledges potential limitations of this approach as dependent variables you analyzed in this study seem likely to be highly related, as they represent different aspects of patient care. Perhaps justify this decision by clearly demonstrating in the methods or **discussion sections** why the dependent variables are truly independent. Without such justification, the current statistical approach remains a significant concern.
