# Supplementary material for: Accessibility and quality of care for adults with hypertension in rural Burkina Faso: results from a cross-sectional household survey
Source: PLOS Glob Public Health. 2025 Apr 2;5(4):e0003161. doi: 10.1371/journal.pgph.0003161 (PMC11964235; doi:10.1371/journal.pgph.0003161)
Supplement: Attachment — Submitted filename: Response to reviewers_Nov24.docx [file pgph.0003161.s020.docx]

Response to reviewers

Dear reviewers,

I would like to thank the editor and reviewers for reviewing this paper. Please find our response to each comment below.

Kind regards

Stephanie Lacey

**Editor**

Please review your reference list to ensure that it is complete and correct. If you have cited papers that have been retracted, please include the rationale for doing so in the manuscript text, or remove these references and replace them with relevant current references. Any changes to the reference list should be mentioned in the rebuttal letter that accompanies your revised manuscript. If you need to cite a retracted article, indicate the article's retracted status in the References list and also include a citation and full reference for the retraction notice.

**Reply:** We have checked the reference list and can confirm no retracted articles are included in this manuscript. Upon review two citation changes have been made to ensure original articles are cited.

Please see track changes page 29, lines 539-543; and page 30, lines 560-565.

In text:

*“WHO. Global Status Report on Noncommunicable Diseases 2010. World Health Organization; Geneva: 2011”.*

*“Shahu A, Herrin J, Dhruva SS, Desai NR, Davis BR, Krumholz HM, Spatz ES. Disparities in Socioeconomic Context and Association With Blood Pressure Control and Cardiovascular Outcomes in ALLHAT. J Am Heart Assoc. 2019;8(15):e012277”.*

**Reviewer 2**

Thank you for your detailed response to my previous feedback, I appreciate the authors’ efforts. Most of my concerns were fully addressed, but I recommend the following:

**Point 1:** Please revise the term “odds ratios” to “prevalence odds ratios.” While the calculation is the same for both, “prevalence odds ratios” is more accurate for a cross-sectional study design, and will help avoid confusion for readers with epidemiological expertise.

**Reply:** We have done as suggested and changed the term odds ratio to prevalence odds ratio throughout the manuscript. At first mention of the term prevalence odds ratio we have indicated that this is equivalent to odds ratio for the benefit of clinicians who may be unfamiliar with the term prevalence odds ratio.

Please see track changes page 9, lines 216-218, as well as minor term amends throughout the manuscript and supplementary tables.

In the text:

*“Results for logistic regression models are presented as prevalence odds ratio (POR, equivalent to odds ratio). Results for linear regression models are presented as regression coefficients”.*

**Point 2:** Since you have chosen the use of multivariable regression models in this context, your rebuttal notwithstanding, I recommend you include a statement that acknowledges potential limitations of this approach as dependent variables you analyzed in this study seem likely to be highly related, as they represent different aspects of patient care. Perhaps justify this decision by clearly demonstrating in the methods or discussion sections why the dependent variables are truly independent. Without such justification, the current statistical approach remains a significant concern.

**Reply:** We have addressed this by including a statement of acknowledgement in the limitations.

Please see tracked changes page 27, lines 496-498.

In the text:

*“Some of the variables used in the multivariable analysis may be colinear, however, substantial co-linearity was excluded prior to conducting the multivariable analyses by ensuring the VIF was low”.*
