# Supplementary material for: Accessibility and quality of care for adults with hypertension in rural Burkina Faso: results from a cross-sectional household survey
Source: PLOS Glob Public Health. 2025 Apr 2;5(4):e0003161. doi: 10.1371/journal.pgph.0003161 (PMC11964235; doi:10.1371/journal.pgph.0003161)
Supplement: Attachment — Submitted filename: Review-PGPH-24-00560-R1-01-04-2025.docx [file pgph.0003161.s019.docx]

Thank you for additional revisions to the manuscript following my previous feedback. The authors acknowledged the flaw in using “odds ratio” for this cross-sectional study and adopted the more fitting “prevalence odds ratio.”

The authors have to some extent acknowledged the limitations of cross-sectional study design using mostly self-reported variables to measure a broad range of patient-centeredness outcomes. Depending on the journal’s requirements, a section on study limitations would have been more ideal but this should not affect the final editorial decision on manuscript publication.
